# Aptamer‐Targeted PrPC Drives Colorectal Cancer Metastasis via a LYN‐STAT3 Complex and Enables Liquid Biopsy Detection

**DOI:** 10.1002/advs.75758

**Published:** 2026-05-19

**Authors:** Chunlin Wang, Hongyu Wu, Chaojing Zheng, Hao Zhang, Xiaoqiu Wu, Jiaqi Wang, Zewen Chang, Jun Xiang, Yunxiao Liu, Chenkai Zhang, Yuliuming Wang, Hao Jiang, Yuchen Zhong, Jun Luo, Ying Chen, NaNa Zhang, Weiyuan Zhang, Ziming Yuan, ChaoXia Zou, Weihong Tan, Meng Wang, Hanqing Hu, Tao Bing, Guiyu Wang

**Affiliations:** ^1^ Department of Colorectal Cancer Surgery The Second Affiliated Hospital of Harbin Medical University Harbin Heilongjiang P. R. China; ^2^ The Key Laboratory of Zhejiang Province for Aptamers and Theranostics Zhejiang Cancer Hospital Hangzhou Institute of Medicine (HIM) Chinese Academy of Sciences Hangzhou Zhejiang P. R. China; ^3^ Molecular Science and Biomedicine Laboratory (MBL) State Key Laboratory of Chemo/Biosensing and Chemometrics College of Chemistry and Chemical Engineering College of Biology Aptamer Engineering Center of Hunan Province Hunan University Changsha Hunan P. R. China; ^4^ Department of Thoracic Surgery Fujian Medical University Union Hospital Fuzhou Fujian P. R. China; ^5^ Department of General Surgery The Fourth Affiliated Hospital of Harbin Medical University Harbin Heilongjiang P. R. China; ^6^ Cancer Center Department of Medical Oncology Zhejiang Provincial People's Hospital Affiliated People's Hospital Hangzhou Medical College Hangzhou Zhejiang P. R. China; ^7^ Department of Colorectal Cancer Surgery Zhejiang Cancer Hospital Hangzhou Institute of Medicine (HIM) Chinese Academy of Sciences Hangzhou Zhejiang P. R. China; ^8^ Department of Pathology The Second Affiliated Hospital of Harbin Medical University Harbin Heilongjiang P. R. China; ^9^ Department of Biochemistry and Molecular Biology Harbin Medical University Harbin Heilongjiang P. R. China

**Keywords:** aptamer, colorectal cancer, metastasis, phosphorylation, prion protein

## Abstract

Colorectal cancer (CRC) poses a significant global health challenge, highlighting the need for better diagnostic tools and molecular targets. Compared to traditional antibodies, aptamers, which are single‐stranded oligonucleotides with high affinity and low immunogenicity, offer an ideal platform for discovering novel biomarkers. Based on this approach, we used the Cell‐SELEX strategy to develop a high‐affinity aptamer‐based probe, WHY‐3E, which successfully identified the cellular prion protein (PrPC) as a key molecular target. We observed that PrPC was significantly upregulated in CRC tissues, strongly correlating with unfavorable clinical outcomes. Functionally, PrPC promotes malignant phenotypes, including migration and invasion, by regulating MSN. Mechanistically, we revealed that PrPC physically translocates from the cell surface to the cytoplasm via endocytosis. Once internalized, it interacts with the STAT3‐NTD and LYN‐SH domains, facilitating the ternary complex formation that enhances LYN‐mediated STAT3 phosphorylation, ultimately increasing MSN transcriptional activity. Moreover, the deubiquitinase USP18 stabilizes PrPC by removing its Lys48‐linked ubiquitin chains, ensuring the continuous activation of this oncogenic axis. Notably, the WHY‐3E aptamer achieved 90.6% sensitivity and 89.0% specificity in detecting PrPC‐positive circulating exosomes in patient cohorts. These findings deepen the mechanistic understanding of CRC progression and offer novel strategic avenues for PrPC‐targeted aptamer applications in non‐invasive diagnostics.

## Graphical abstract

The aptamer WHY‐3E identifies PrPC as a CRC driver. Stabilized by USP18, endocytosed PrPC forms a LYN/STAT3 complex, upregulating MSN transcription to promote metastasis. Crucially, WHY‐3E sensitively detects PrPC‐positive circulating exosomes, establishing a robust theoretical foundation for non‐invasive clinical diagnostics.

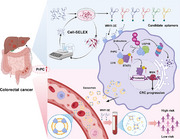

## 1 Introduction

Colorectal cancer (CRC) is a leading cause of cancer‐related mortality worldwide, primarily driven by metastatic dissemination [1, 2]. However, despite significant efforts to understand the molecular drivers of CRC progression, the complex signaling networks that orchestrate metastasis have not been fully elucidated, highlighting the need to identify novel functional hubs within these networks.

Cellular prion protein (PrPC), encoded by the PRNP gene, is a glycosylated membrane protein whose misfolded isoform, PrPSc, has long been associated with the pathogenesis of neurodegenerative spongiform encephalopathies. This has led previous studies to focus predominantly on this pathological form [3, 4]. However, recent evidence indicates that PrPC is vital for the progression of various malignant tumors [5, 6, 7]. PrPC, which is localized to the plasma membrane, functions as a signaling hub [8]. Mechanistically, PrPC binds to amyloid‐β peptide (Aβ), mediating the Aβ‐PrPC‐CAV1 signaling axis, which regulates the mesenchymal phenotype and metastatic potential of prostate and colon cancers [9]. Additionally, PrPC modulates Rac1 activity to promote lamellipodia formation and cell migration by interacting with upstream molecules of the JNK pathway [10]. Furthermore, PrPC colocalizes with EGFR on the plasma membrane, enhancing its dimerization and activation, thereby initiating intracellular transcriptional programs [11]. However, this membrane‐centric view obscures a complex biological reality. Driven by dynamic internalization and intracellular trafficking, this protein is increasingly recognized to localize to the cytoplasm to execute non‐canonical functions [12, 13, 14]. Yet, the functional significance of this intracellular pool and its specific contribution to CRC progression remain largely unexplored, representing a major knowledge gap in the current field.

Aptamers are single‐stranded oligonucleotides, obtained in vitro via the SELEX technique, that specifically bind to their targets. They have emerged as powerful molecular tools owing to their low immunogenicity, high stability, ease of synthesis, and exceptional specificity [15, 16]. A key advancement in this field is the Cell‐Systematic Evolution of Ligands by EXponential Enrichment (SELEX), which selects aptamers using intact living cells under physiological conditions rather than recombinant proteins [17]. This approach enables the direct discovery of binders targeting unknown or poorly characterized cell‐surface molecules, thereby revealing new targets in their native states. Although the aptamer molecular targets obtained through Cell‐SELEX are initially unknown, integrating high‐throughput sequencing and bioinformatics analysis allows systematic target identification, turning this challenge into an opportunity for novel biomarker discovery [18]. These properties make nucleic acid aptamers ideal tools for mapping the cellular surfaceome and probing functional protein networks in living cells. The combination of Cell‐SELEX and sequencing technologies provides a powerful strategy for discovering new biological targets and generating high‐quality aptamers with significant translational potential in diagnostics, molecular imaging, and targeted therapies.

Leveraging this foundation, we uncovered a previously unrecognized intracellular signaling axis that is vital for CRC progression. We demonstrate that PrPC undergoes dynamic internalization to enter the cytoplasm, where it interacts with the Src family kinase LYN and the transcription factor STAT3, forming a critical PrPC‐LYN‐STAT3 ternary complex. This complex drives the metastasis promoter moesin (MSN) transcription. Furthermore, we identified the deubiquitinase USP18 as a key upstream regulator that stabilizes PrPC by removing Lys48‐linked ubiquitination. Collectively, our findings delineate a complete PrPC/LYN/STAT3/MSN signaling circuit and identify USP18 as a novel regulator. Importantly, we revealed that the WHY‐3E aptamer can detect PrPC‐positive circulating exosomes, highlighting its translational potential as a non‐invasive screening tool.

## 2 Results

### 2.1 PrPC was Identified as a CRC Target Associated With Adverse Prognosis

**FIGURE 1:**
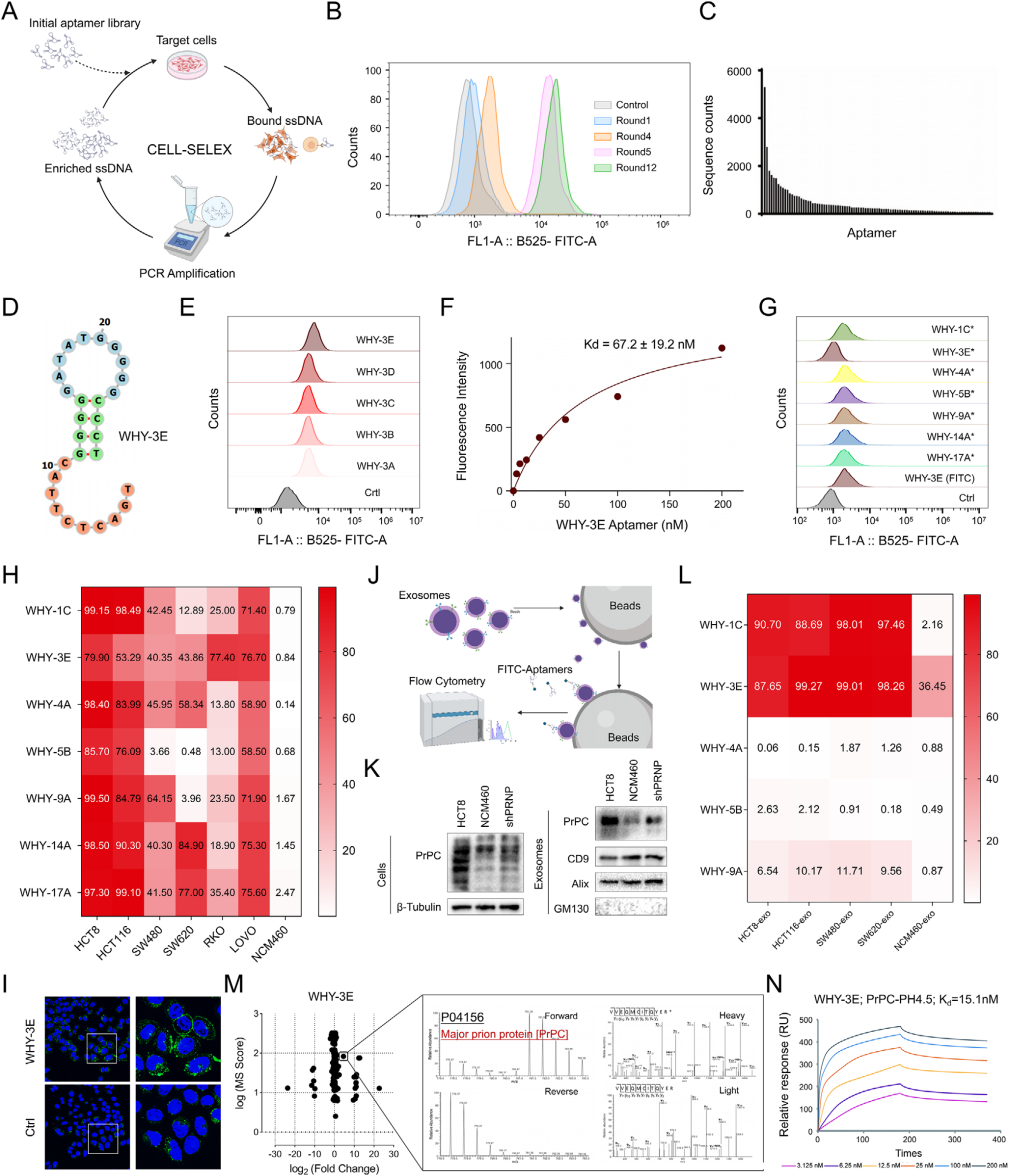
Screening for CRC‐targeting aptamers and identifying the target of aptamer WHY‐3E using Cell‐SELEX. (A) Schematic of the Cell‐SELEX procedure used for the selection of aptamers against HCT8 cells. (B) Flow cytometry analysis demonstrating the enrichment of cell‐binding aptamer pools across selection rounds (R1, R4, R5, R12). (C) Length distribution of the R12 aptamer pool from high‐throughput sequencing. (D) Predicted secondary structure of the WHY‐3E aptamer. (E) Binding capacity of the aptamer WHY‐3E to HCT8 cells, assessed by flow cytometry. (F) Binding affinity curve of WHY‐3E for HCT8 cells, as determined by flow cytometry. The dissociation constant (K_d_) of aptamer WHY‐3E was determined to be 67.2 ± 19.2 nm. (G) Competitive binding analysis among selected aptamers. FAM‐labeled aptamers were used as probes, and their unlabeled counterparts as competitors. (H) Binding specificity of different aptamers to a panel of cell lines (HCT8, HCT116, SW480, SW620, RKO, LOVO) and a normal colonic epithelial cell line (NCM460), presented as a heatmap of positive binding rates. (I) Immunofluorescence staining showing the subcellular binding morphology of FITC‐labeled WHY‐3E (green) in HCT8 cells, with nuclei stained by DAPI (blue). (J) Schematic diagram of aptamer‐bound exosomes detected by flow cytometry. (K) PrPC levels in HCT8 shNC cells, NCM460 cells, and shPRNP cells, as well as in their derived exosomes. CD9 and Alix were used as exosomal biomarkers, while GM130 served as a negative marker. (L) Binding specificity of selected aptamers to exosomes from different cell lines, presented as a heatmap of fluorescence intensity. (M) Identification of the WHY‐3E target protein through LC‐MS/MS analysis, with candidate proteins ranked by fold change and MS score. (N) Surface plasmon resonance (SPR) sensorgrams showing the direct binding of WHY‐3E to recombinant PrPC protein at increasing concentrations (3.125, 6.25, 12.5, 25, 100, 200 nm).

To develop a robust tool for probing the molecular signatures of CRC cells, we used Cell‐SELEX to screen for high‐affinity aptamers against CRC cells (Figure 1A). The fluorescence intensity of the ssDNA library binding to HCT8 cells gradually increased from round 1 (R1) to round 4 (R4), followed by a marked increase at round 5 (R5), after which binding intensity reached a plateau with only a modest increase through round 12 (R12) (Figure 1B). High‐throughput sequencing of the R12 product identified 53558 unique sequences, and the top 100 sequences from R12 were ranked based on their relative abundance (Figure 1C). Based on molecular abundance and secondary structure characteristics, we selected seven candidate aptamers and empirically performed truncation modification [19, 20]. The secondary structures of the aptamers were predicted using mFold [21] (Figure 1D and Figure S1). Their binding capacity to CRC cell lines was then evaluated using flow cytometry. The results revealed that seven of these subsequences, designated WHY‐1C, WHY‐3E, WHY‐4A, WHY‐5B, WHY‐9A, WHY‐14A, and WHY‐17A, exhibited strong binding affinity (Figure 1E and Figure S2). The K_d_ values of these seven aptamers were further determined, ranging from 12.1 to 84.9 nm (Figure 1F and Figure S3). To assess whether different aptamers share overlapping binding sites, we performed competitive binding assays. FAM‐labeled aptamers (200 nm) were incubated with HCT8 cells in the presence of unlabeled aptamers (2 µm) as competing ligands. A reduction in fluorescence intensity indicated competition for shared binding sites. The competition analysis revealed significant competition among WHY‐4A, WHY‐14A, and WHY‐17A in HCT8 cells, with WHY‐4A exhibiting the strongest binding affinity (Figure 1G and Figure S4). Finally, binding specificity was further assessed by heatmap analysis, which demonstrated that five aptamers – WHY‐1C, WHY‐3E, WHY‐4A, WHY‐5B, and WHY‐9A – specifically bound to CRC cells, with minimal binding to normal colonic epithelial cells (Figure 1H). Furthermore, immunofluorescence (IF) assays demonstrated that the candidate aptamers exhibited strong binding capacity and specificity to CRC cells (Figure 1I and Figure S5A).

Previous studies have confirmed that exosomes act as carriers to mediate the transport of oncogenic molecules, thereby remodeling the tumor microenvironment [22]. Thus, the detection of tumor‐derived exosomes has emerged as a highly promising diagnostic strategy for liquid biopsy in the biomedical field [23]. Given the potential application value of aptamers in the targeted detection of tumor‐derived exosomes, the present study further aimed to screen for candidate aptamers that can specifically bind to tumor cell‐derived exosomes. To this end, exosomes were immobilized onto aldehyde/sulfate latex beads, and after co‐incubation with the candidate aptamers, the binding capacity of the aptamers to exosomes was evaluated by flow cytometry (Figure 1J). The isolated exosomes were characterized as typical exosomes by Western blot (positive for CD9 and Alix; negative for GM130), transmission electron microscopy (TEM, showing typical cup‐shaped morphology), and nanoparticle tracking analysis (NTA, revealing an average particle size of approximately 87 nm) (Figure 1K and Figure S5B). Exosomes were further isolated from HCT8 cells transfected with shNC or PRNP‐targeting shRNA, as well as from NCM460 cells. The results showed that PrPC was expressed in both HCT8 cells and their derived exosomes, and knockdown of PrPC in cells led to a corresponding reduction in exosomal PrPC levels (Figure 1K). Exosome binding assays and heatmap analysis of fluorescence intensity revealed that among the candidate aptamers with favorable cell binding properties, only WHY‐1C and WHY‐3E exhibited strong and specific binding to CRC cell‐derived exosomes (Figure 1L). Collectively, WHY‐1C and WHY‐3E were the only aptamers that demonstrated optimal binding affinity and specificity at both the CRC cells and tumor‐derived exosomes, and were therefore selected for subsequent molecular target identification.

Based on these findings, we used a SILAC‐based proteomic approach to identify the molecular target of the high‐affinity aptamers WHY‐1C and WHY‐3E. CRC cells were metabolically labeled and incubated with biotin‐conjugated aptamers. Proteins bound to the aptamer were captured using streptavidin‐coated agarose beads and subsequently identified using liquid chromatography‐tandem mass spectrometry (LC‐MS/MS) [24]. This sequence analysis initially identified Epithelial Cell Adhesion Molecule (EpCAM) as the candidate target of WHY‐1C and cellular prion protein (PrPC) as that of WHY‐3E (Figure 1M). As a well‐established classic biomarker, EpCAM has been extensively documented in terms of its biological functions, and regulatory mechanisms [25, 26]. In contrast, PrPC is a highly conserved glycoprotein involved in the regulation of normal physiological activities. Previous studies have shown that it is highly expressed in CRC, but its biological functions, molecular mechanisms, and translational potential remain unclear [27]. To further validate the sequencing results, we performed SPR analysis using purified recombinant PrPC protein. The results demonstrated that WHY‐3E binds PrPC with high affinity (K_d_ = 15.1 nm) (Figure 1N). Subsequent aptamer pull‐down assays independently confirmed the specific interaction between WHY‐3E and PrPC (Figure S5C). Meanwhile, by downregulating cellular PrPC levels, the binding ability of WHY‐3E to CRC cells was decreased (Figure S5D,E). Collectively, these findings indicate that WHY‐3E specifically recognizes and binds PrPC. Therefore, we selected WHY‐3E and its target PrPC as the focus of subsequent in‐depth investigation, aiming to elucidate their functional roles and underlying mechanisms in CRC progression.

To assess the significance of PrPC in CRC, we first analyzed PrPC expression in eight paired tumors and adjacent non‐tumor tissue samples using WB, which revealed significantly elevated PrPC levels in tumor tissue cells (Figure S6A,B). Furthermore, we established two CRC patient cohorts (an internal cohort and a tissue microarray cohort) and performed IHC staining. The results revealed higher PrPC expression in tumor tissues than in adjacent non‐tumor tissues. Moreover, PrPC expression was significantly elevated in liver metastasis samples from stage IV patients than their primary tumor samples and was positively correlated with the pathological stage (Figure S6C–G). Multivariate Cox regression analysis of patients with cancer from the GSE40967 cohort further confirmed that high PRNP expression is an independent risk factor for mortality (*p* < 0.0001) (Figure S6H). Kaplan–Meier curve analysis revealed that patients with high PRNP expression had significantly shorter overall survival (OS) (Figure S6I,J). Collectively, these findings indicate that PrPC promotes CRC progression and is associated with adverse clinical outcomes.

### 2.2 PrPC Promoted CRC Cell Metastasis In Vitro and In Vivo

**FIGURE 2:**
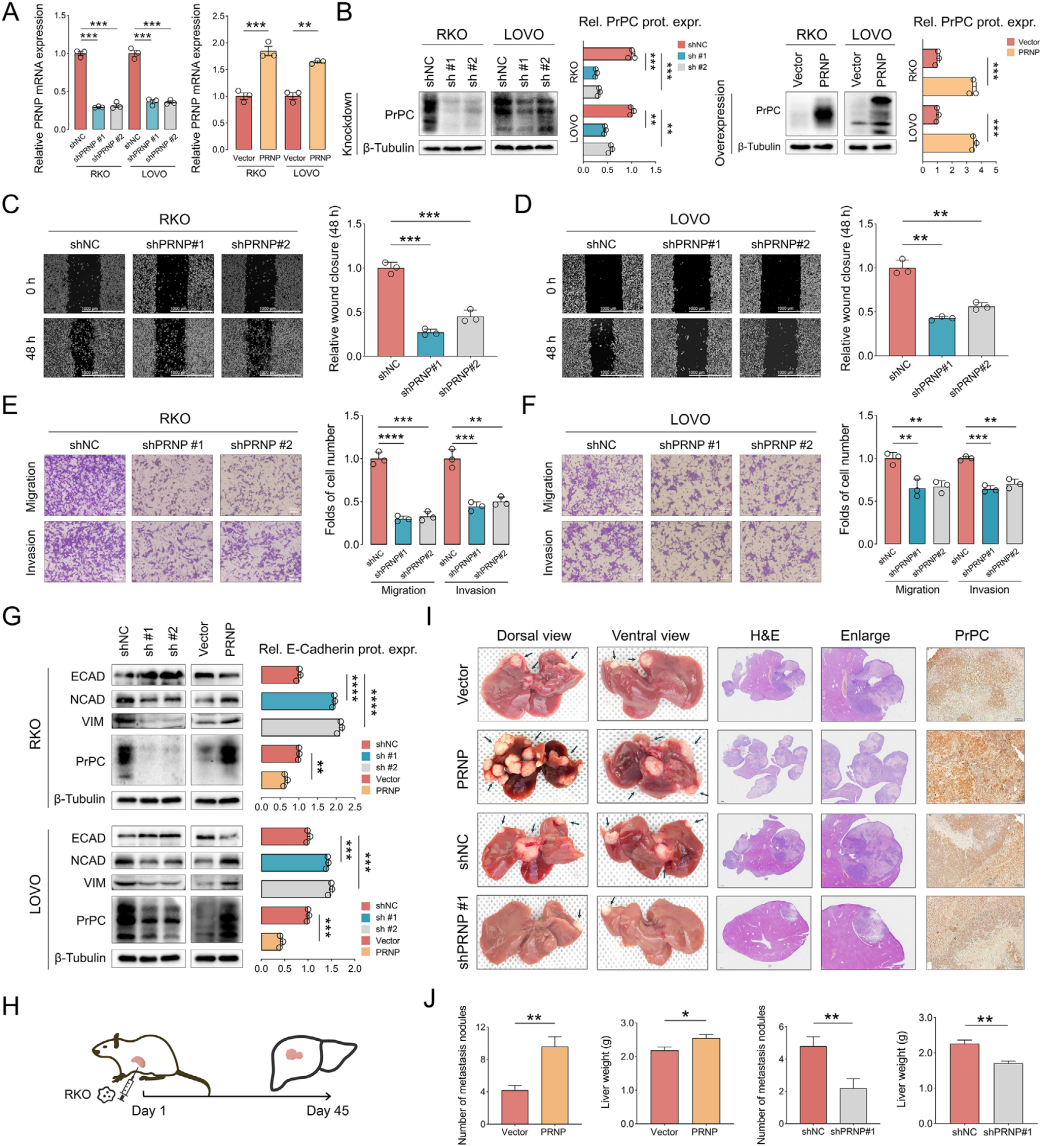
PrPC promotes metastasis of colorectal cancer in vitro and in vivo. (A) RT‐qPCR analysis of PRNP transcript levels in RKO and LOVO cells following knockdown or overexpression, normalized to GAPDH. (B) Western blot analysis of PRNP protein expression in RKO and LOVO cells after knockdown or overexpression, using β‐tubulin as a loading control. (C,D) Wound healing assays evaluating the migratory capacity of RKO and LOVO cells after PRNP knockdown. Representative images (left) and quantitative analysis (right) are presented. (E,F) Transwell assays assessing the migration and invasion capabilities of RKO and LOVO cells upon PRNP knockdown. Representative images (left) and corresponding quantification (right) are shown. (G) Western blot analysis of epithelial‐mesenchymal transition (EMT) marker expression in RKO and LOVO cells following PRNP knockdown or overexpression, using β‐tubulin as a loading control. (H) Schematic of the splenic injection‐liver metastasis model. Nude mice were intrasplenically injected with RKO cells (5 × 10^6^ per mouse) and metastatic liver tumors were analyzed 6 weeks post‐injection. (I) Representative images of liver sections from each group, showing metastatic nodules (left), H&E staining (middle), and immunohistochemical (IHC) staining for PrPC (right) (*n* = 5 mice per group). (J) Statistical analysis of the number of liver metastatic nodules and liver weights from the in vivo metastasis model. Data are representative of at least three independent experiments and presented as mean ± SD. ^*^ *p* < 0.05, ^**^ *p* < 0.01, ^***^ *p* < 0.001, ^****^ *p* < 0.0001.

To study the PrPC biological function, we modulated PrPC expression using two PRNP‐specific shRNAs (shPRNP#1 and shPRNP#2) and PRNP cDNA using lentivirus‐mediated delivery into LOVO and RKO cells. Successful PrPC expression level modulation was confirmed through RT‐qPCR and WB (Figure 2A,B). CCK‐8 and colony formation assay were conducted to measure cell proliferative capability, and the results revealed no trends in several groups (Figure S7A,B). Cell migration and invasion capacity were evaluated using Transwell and wound healing assays. The results demonstrated that PRNP knockdown resulted in a reduction in cell migration and invasion; however, PrPC overexpression led to an enhancement of these properties in RKO and LOVO cells (Figure 2C–F and Figure S7C–F). An extensive study has been undertaken to comprehend the metastasis of epithelial‐origin cancer, particularly focusing on investigating EMT [1, 28]. Therefore, we used WB to examine the EMT‐associated marker expression and observed that PrPC depletion in RKO and LOVO cells resulted in a decrease in N‐cadherin and Vimentin; however, there was an increase in E‐cadherin. Conversely, PRNP overexpression had opposite effects on the protein levels of N‐cadherin, Vimentin, and E‐cadherin (Figure 2G).

To study the PrPC effect on CRC metastasis in vivo, we established a liver metastasis model (Figure 2H). RKO cells from the four groups (shNC, shPRNP#1, Vector, and PRNP) were intravenously injected into nude mice via the splenic vein. The histopathological features of the liver tissue samples obtained from this mouse model were revealed by H&E staining (Figure 2I). Besides, the shPRNP#1 group exhibited a significant reduction in the number of metastatic tumor nodules in the livers of nude mice and liver weight, compared to the shNC group. Conversely, the number of liver metastases and affected liver weight in the PRNP group were significantly higher than those in the control group, significantly (Figure 2J). Collectively, our findings strongly suggest that high PrPC expression promotes CRC cell metastasis in vitro and in vivo.

### 2.3 PrPC Promoted CRC Cell Metastasis by Upregulating MSN

**FIGURE 3:**
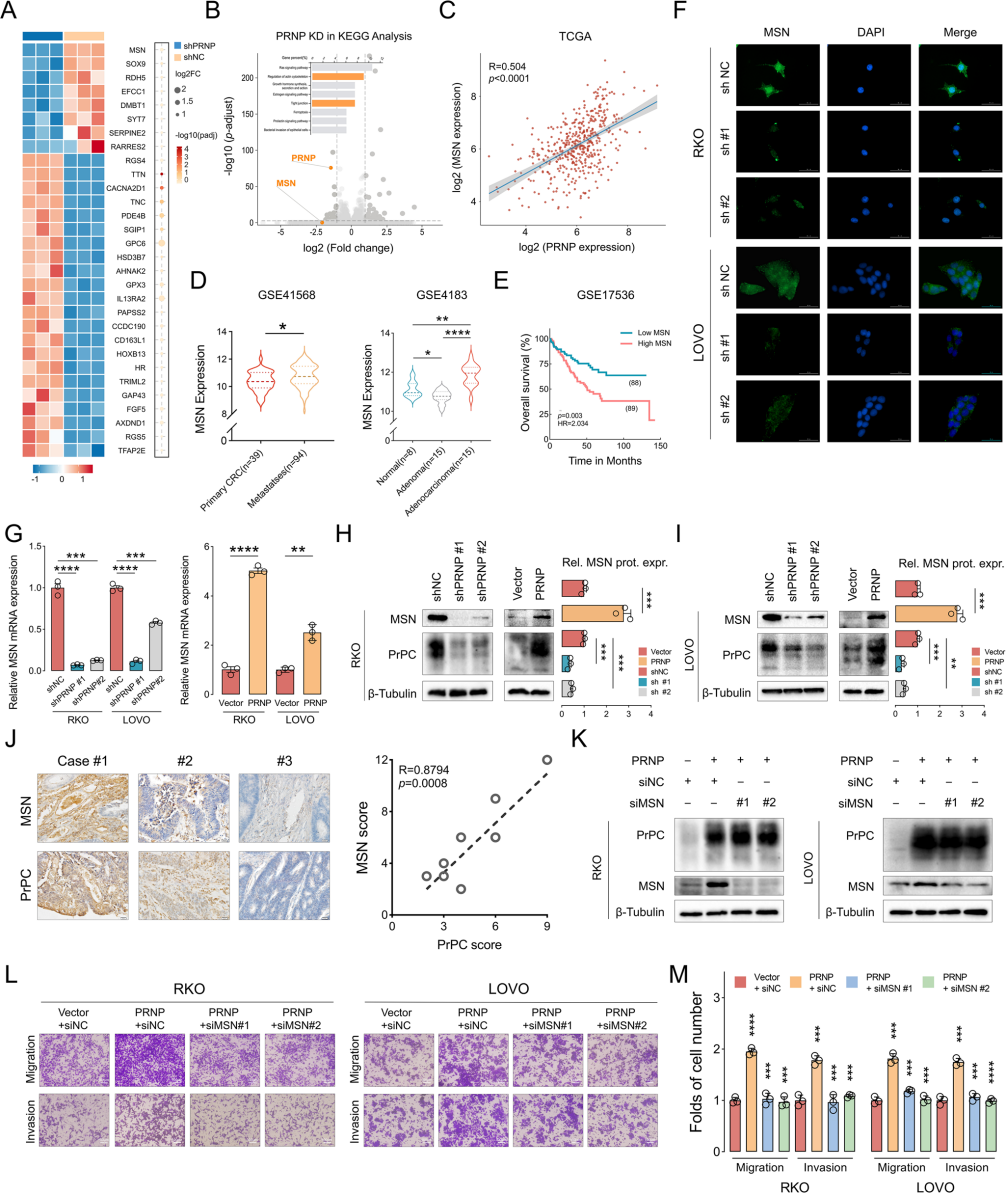
MSN expression is positively correlated with PrPC as its downstream effector. (A) Heatmap displaying 30 dysregulated proteins (|Log_2_FC| > 1.5) identified by transcriptomic analysis of shPRNP RKO cells (*n* = 3 biological replicates per group). (B) Volcano plot of differentially expressed genes from RNA‐seq of shPRNP vs. control cells. Bar graph shows significantly enriched KEGG pathways (*p* < 0.05). (C) Scatter plot illustrating the correlation between PRNP and MSN mRNA expression in the TCGA colorectal cancer cohort. (D) Analysis of PRNP mRNA expression in public datasets GSE41568 and GSE4183, comparing colorectal adenoma, carcinoma, and normal mucosa tissues. (E) Kaplan–Meier analysis of overall survival in CRC patients stratified by high vs. low MSN mRNA expression (GSE17536 dataset). (F) Immunofluorescence analysis of MSN expression in RKO and LOVO cells after PRNP knockdown or overexpression. Scale bar: 30 µm. (G) RT‐qPCR analysis of MSN mRNA levels following PRNP knockdown or overexpression, normalized to GAPDH. (H,I) Western blot analysis of MSN protein expression after PRNP knockdown (H) or overexpression (I), using β‐tubulin as a loading control. (J,K) Representative immunohistochemistry (IHC) images (left) and correlation analysis (right) of PrPC and MSN expression in CRC clinical tissues. (L) Western blot analysis validating the efficacy of MSN knockdown in PRNP‐overexpressing RKO and LOVO cells, with β‐tubulin as a loading control. (M,N) Transwell migration and invasion assays demonstrating that MSN knockdown rescues the enhanced metastatic capacity induced by PRNP overexpression. Data are representative of three independent experiments and presented as mean ± SD. ^*^ *p* < 0.05, ^**^ *p* < 0.01, ^***^ *p* < 0.001, ^****^ *p* < 0.0001.

To delineate the molecular mechanism by which PrPC regulates CRC progression, RNA sequencing (RNA‐seq) with three biological replicates was performed on RKO cells expressing shNC or shPRNP#1. Differentially expressed genes were filtered using thresholds of |log_2_(fold change)| > 1.5 and adjusted *p* < 0.05. MSN emerged as the most significantly downregulated molecule (log_2_FC = −2.05, adjusted *p* < 0.0001) and was robustly enriched in the pathways of “regulation of actin cytoskeleton” and “tight junction” (Figure 3A,B). Database analyses confirmed positive correlation between MSN and PRNP expression (R = 0.504, *p* < 0.0001), significant enrichment in metastatic lesions (*p* < 0.05), and association with poor prognosis (HR = 2.034, *p* = 0.003) (Figure 3C–E). IF assays revealed reduced MSN protein levels upon PRNP knockdown; consistently, PRNP knockdown or overexpression in RKO and LOVO cells coordinately decreased or increased MSN expression at the mRNA and protein levels (Figure 3F–I). Subsequently, we validated the strong positive correlation between MSN and PrPC in 10 tumor specimens using IHC staining (R = 0.8794, *p* = 0.0008) (Figure 3J).

To determine whether PrPC promotes CRC metastasis in an MSN‐dependent manner, we performed rescue experiments. We first transfected PRNP‐overexpressing cells with siMSN or siNC and confirmed efficient MSN knockdown by WB (Figure 3K). Functional assays demonstrated that MSN silencing effectively rescued the pro‐metastatic effects of PrPC. Specifically, MSN knockdown significantly suppressed the enhanced migration and invasion of PRNP‐overexpressing cells compared to the siNC controls (*p* < 0.001 for both cell lines) (Figure 3L,M). These findings establish MSN as a critical downstream effector through which PrPC promotes migration and invasion in CRC.

### 2.4 PrPC Regulated MSN by Activating the STAT3 Phosphorylation

**FIGURE 4:**
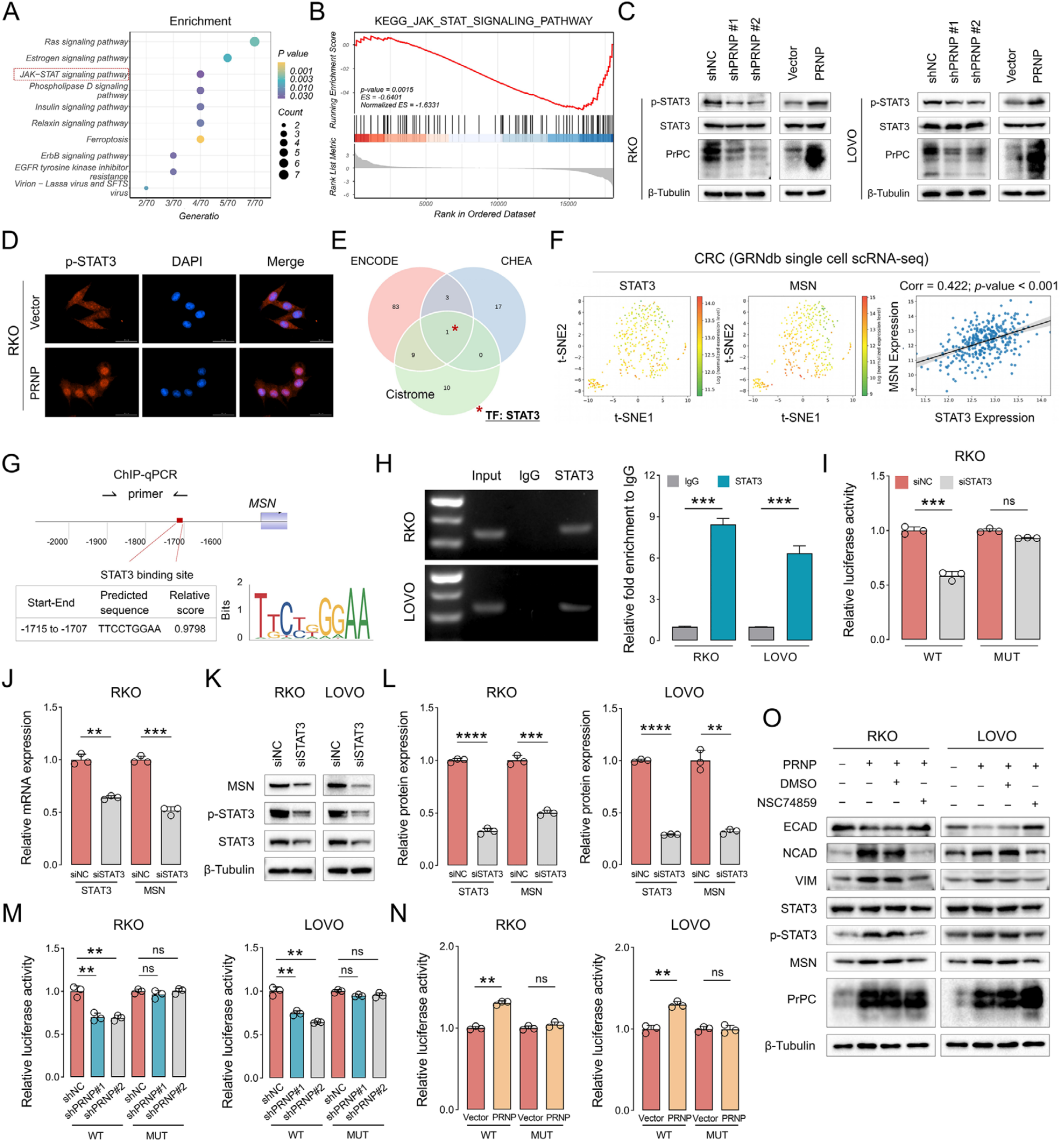
PrPC regulates MSN expression via STAT3 phosphorylation. (A) Bubble plot of KEGG pathway enrichment analysis for downregulated genes from RNA‐seq of shPRNP RKO cells. (B) Gene set enrichment analysis (GSEA) plot showing significant suppression of the JAK‐STAT signaling pathway in PRNP‐knockdown RKO cells (*n* = 3). (C) Western blot analysis of STAT3 and p‐STAT3 (Tyr705) levels in RKO and LOVO cells following PRNP modulation, using β‐tubulin as a loading control. (D) Immunofluorescence staining showing PrPC‐induced nuclear translocation of p‐STAT3 (red) in RKO cells. Nuclei were counterstained with DAPI (blue). Scale bar: 30 µm. (E) Venn diagram identifying STAT3 as a candidate transcription factor for MSN by intersecting predictions from the CHEA, CISTROME, and ENCODE databases. (F) Feature plots and a scatter plot depicting the correlation between STAT3 and MSN expression in the TCGA colorectal cancer single‐cell transcriptome dataset. (G) Prediction of STAT3 binding motifs in the MSN promoter region using the NCBI and JASPAR database. Schematic illustrates the top candidate binding site. (H) Chromatin immunoprecipitation (ChIP)‐qPCR assay quantifying STAT3 enrichment at the MSN promoter in RKO cells. (I) Dual‐luciferase reporter assay measuring MSN promoter activity in RKO cells with or without STAT3 knockdown. Data are normalized to Renilla luciferase activity. (J) RT‐qPCR analysis of MSN mRNA expression after STAT3 knockdown in RKO cells, normalized to GAPDH. (K,L) Western blot analysis of MSN protein levels after STAT3 knockdown in RKO and LOVO cells, using β‐tubulin as a loading control. (M‐N) Dual‐luciferase reporter assay measuring STAT3 transcriptional activity in RKO and LOVO cells with PRNP knockdown (M) or overexpression (N). (O) Western blot analysis of EMT markers, p‐STAT3, and MSN expression in PRNP‐overexpressing cells treated with the STAT3 inhibitor NSC74859, using β‐tubulin as a loading control. Data are representative of three independent experiments and presented as mean ± SD. ^**^ *p* < 0.01, ^***^ *p* < 0.001, ^****^ *p* < 0.0001.

Previous studies have established involvement of PrPC in MSN transcriptional regulation; however, the underlying mechanism remains elusive. Further integrated analysis of RNA‐seq data, using KEGG functional enrichment and Gene Set Enrichment Analysis (GSEA), suggested a potential positive correlation between PRNP and STAT3‐targeted signaling pathway (Figure 4A,B). Experimental validation in PRNP‐overexpressing cells demonstrated markedly enhanced STAT3 phosphorylation (Tyr705) and significant pathway activation. Conversely, this effect was reversed by PRNP knockdown (Figure 4C). Subcellular fractionation and immunofluorescence assays in RKO and LOVO cells demonstrated that PRNP overexpression specifically enhanced the nuclear accumulation, but not the cytoplasmic distribution, of phosphorylated‐STAT3 (p‐STAT3) (Figure 4D and Figure S8A,B). Taken together, these findings indicate that PrPC exerts its oncogenic function by activating STAT3 signaling.

Subsequently, interrogation of major transcription factor databases (ENCODE, Cistrome, and CHEA) consistently identified STAT3 as the primary transcriptional regulator of MSN (Figures 4E and Figure S8C). Consequently, we proposed that PrPC upregulates MSN transcription by enhancing STAT3 phosphorylation (Tyr705). This hypothesis was supported by deep scRNA‐seq analysis of the GRNdb database, which confirmed a strong positive correlation between STAT3 and MSN expression (R = 0.422; *p* < 0.001) (Figure 4F).

To identify putative transcription factor binding sites within the MSN promoter, we performed JASPAR analysis, which revealed STAT3 binding motifs. A predicted site was located at positions between –1715 and –1707 (TTCCTGGAA) upstream of the MSN transcription start site (Figure 4G). To determine whether STAT3 directly activates MSN transcription in CRC cells, we conducted ChIP‐qPCR assays, which confirmed STAT3 occupancy of the MSN promoter (Figure 4H). We validated these predictions using luciferase reporter assays with wild‐type (WT) and mutated (MUT) MSN promoters (containing altered putative STAT3 binding sites). STAT3 knockdown significantly reduced luciferase activity, respectively, in RKO and LOVO cells transfected with the MSN‐promoter‐WT construct, while not affecting the MUT construct (Figure 4I and Figure S8D). Subsequent qPCR and WB analyses confirmed that STAT3 knockdown or overexpression decreased or increased MSN mRNA and protein levels, respectively, in both cell lines (Figure 4J–L and Figure S8E). Given that PrPC activates STAT3 and promotes its nuclear translocation, we assessed whether PrPC influences MSN transcription by measuring STAT3‐dependent luciferase promoter activity. Dual‐luciferase assays demonstrated that PRNP knockdown significantly reduced MSN transcriptional activity in RKO and LOVO cells, whereas PRNP overexpression enhanced it. Critically, PrPC modulation did not affect MSN promoter activity, which harbors mutant STAT3 binding sites (Figure 4M,N). To further validate that PrPC regulates MSN activity through the STAT3 pathway, we treated PRNP‐overexpressing cells with or without the STAT3 inhibitor, NSC74859. We observed that NSC74859 reversed the PRNP overexpression‐induced enhancements in EMT markers, p‐STAT3, and MSN expression upregulation (Figure 4O). Furthermore, this treatment significantly inhibited the enhanced cell migration and invasion capabilities induced by PRNP overexpression (Figure S8F,G). These results provide compelling evidence that PrPC positively regulates MSN transcriptional activity by activating the STAT3 pathway and promoting phosphorylation and nuclear accumulation.

### 2.5 The Dynamic Internalization of PrPC Drives Intracellular Signal Activation

The spatial discrepancy between the cell‐surface localization of PrPC and the intracellular activation of STAT3 prompted us to investigate how this membrane protein triggers downstream signaling. Although PrPC is classically defined as a GPI‐anchored surface protein, we reasoned that its cytoplasmic accumulation might result from a dynamic spatial transition. To test this, we first monitored the physical movement of membrane‐bound PrPC using a cleavable cell‐surface biotinylation assay (Figure S9A,B). In both CRC cells, surface‐labeled PrPC was internalized and accumulated within the intracellular pool in a time‐dependent manner (0–60 min). To determine whether this internalization is indeed the prerequisite for signaling initiation, we employed Dynasore to block Dynamin‐mediated endocytosis (Figure S9C–F). Subcellular fractionation showed that Dynasore treatment led to a significant retention of PrPC on the plasma membrane, with a concurrent depletion of the cytoplasmic PrPC pool. Crucially, this pharmacological blockade of endocytosis markedly attenuated STAT3 phosphorylation at Tyr705 (Figure S9G,H). These results suggest that PrPC may undergo dynamic internalization to replenish its cytosolic reservoir, a process that is indispensable for its subsequent interaction with and activation of the STAT3 signaling cascade.

### 2.6 PrPC Simultaneously Interacted With LYN and STAT3 to Form a Ternary Complex

**FIGURE 5:**
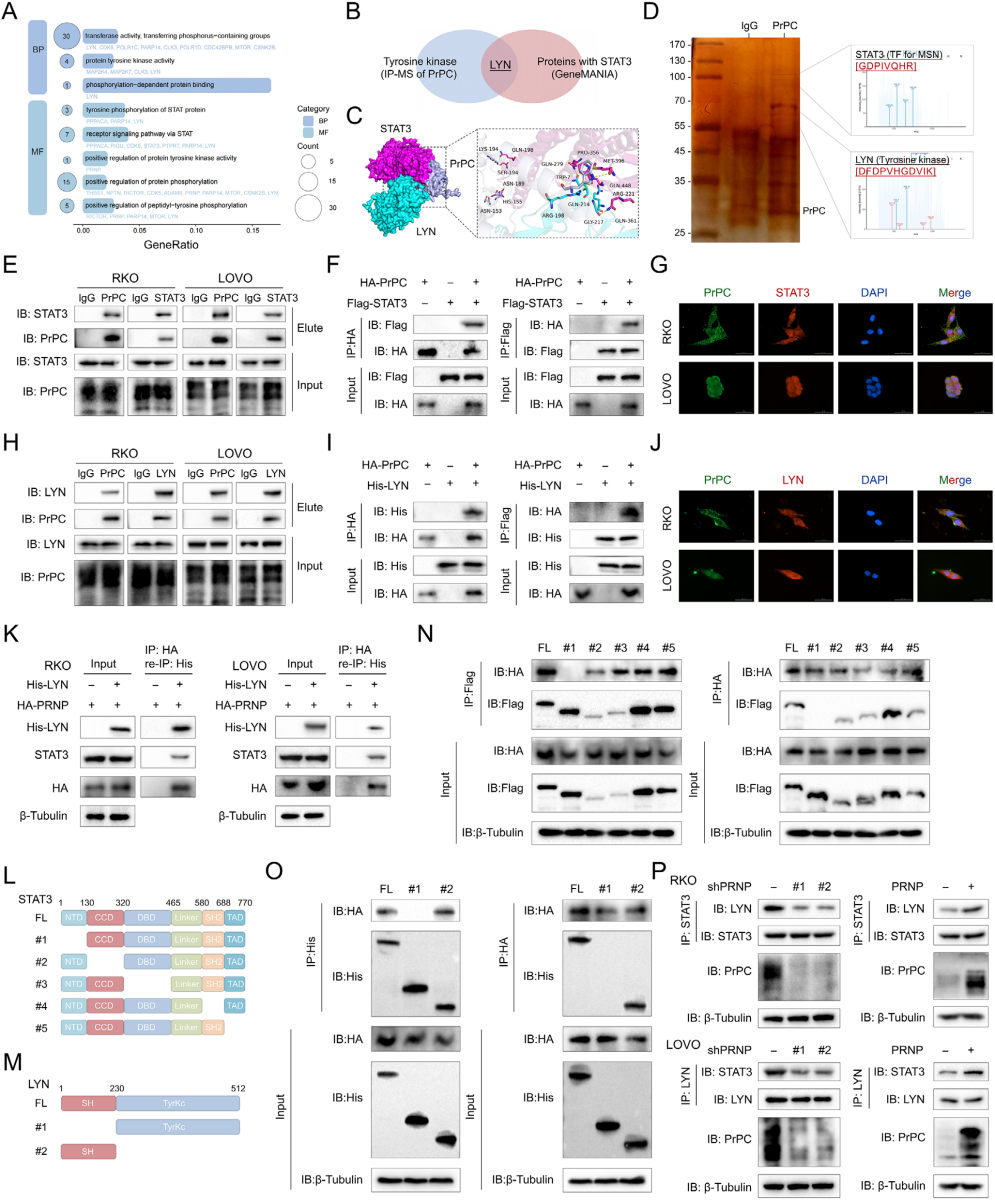
PrPC simultaneously interacted with LYN and STAT3 to form a ternary complex. (A) Significantly enriched Gene Ontology (GO) terms for proteins identified in the PrPC immunoprecipitation‐mass spectrometry (IP‐MS) experiment. (B) Venn diagram identifying the non‐receptor tyrosine kinase LYN as the shared interactor between PrPC and STAT3 protein networks. (C) Computational protein–protein docking model predicting the binding interface among PrPC, LYN, and STAT3, with hydrogen bonds identified as a key stabilizing interaction. (D) Silver‐stained gel after immunoprecipitation with an anti‐PrPC antibody. Arrows indicate specific protein bands at expected molecular weights. (E) Endogenous co‐immunoprecipitation (Co‐IP) confirming the interaction between PrPC and STAT3 in RKO and LOVO cell lysates. (F) Exogenous Co‐IP validating the PrPC‐STAT3 interaction in RKO and LOVO cells transfected with tagged constructs. (G) Immunofluorescence (IF) images showing co‐localization of PrPC (green) and STAT3 (red) in the cytoplasm of RKO and LOVO cells. Nuclei were stained with DAPI (blue). Scale bar: 30 µm. (H) Endogenous Co‐IP confirming the interaction between PrPC and LYN in RKO and LOVO cells. (I) Exogenous Co‐IP validating the PrPC‐LYN interaction in transfected RKO and LOVO cells. (J) IF images showing co‐localization of PrPC (green) and LYN (red) in the cytoplasm. Nuclei were stained with DAPI (blue). Scale bar: 30 µm. (K) IP‐re‐IP assay confirming the formation of a ternary complex among PrPC, LYN, and STAT3 in CRC cells. (L) Domain architecture of STAT3 truncation mutants. NTD, N‐terminal domain; CCD, coiled‐coil domain; DBD, DNA‐binding domain; SH2, Src homology 2 domain; TAD, transactivation domain. (M) Domain architecture of LYN truncation mutants. (N) Co‐IP analysis identifying the NTD domain of STAT3 as critical for its binding to PrPC. (O) Co‐IP mapping analysis identifying the SH domain of LYN as the primary region responsible for binding to PrPC. (P) Co‐IP assays reveal that PRNP overexpression enhances, while its knockdown diminishes, the interaction between endogenous LYN and STAT3 in RKO and LOVO cells. Data are representative of three independent experiments and presented as mean ± SD.

These findings prompted us to further study the specific molecular mechanism by which intracellular PrPC activates the STAT3 pathway. Using immunoprecipitation mass spectrometry (IP‐MS), we identified PrPC‐interacting proteins. Subsequent Gene Ontology (GO) enrichment analysis revealed a significant association between PrPC and the regulation of protein phosphorylation pathways (Figure 5A). Although PrPC itself is not a kinase, we hypothesized that it might enhance the interaction between a tyrosine kinase and STAT3, thereby promoting STAT3 phosphorylation. Among the tyrosine kinases that co‐immunoprecipitated with PrPC, cross‐referencing with the GENEMANIA database of STAT3‐interacting proteins identified LYN as the sole overlapping candidate (Figure 5B). LYN, a member of the Src family of tyrosine kinases, was functionally enriched in pathways associated with STAT phosphorylation and transferase activity involving phosphoryl groups [29] (Figure 5A). Based on these observations, we proposed that PrPC, LYN, and STAT3 form a ternary complex. To further elucidate the spatial binding mechanism among these proteins, we performed protein–protein molecular docking, which yielded a docking score of −262.38, supporting stable ternary complex formation (Figure 5C and Figure S10A). Subsequent validation using silver staining of co‐immunoprecipitated proteins confirmed that PrPC interacted simultaneously with STAT3 and LYN (Figure 5D).

Subsequently, we sought to delineate the molecular interactions between PrPC, STAT3, and LYN. Co‐IP assays confirmed that PrPC binds to STAT3 in CRC cells under exogenous overexpression and endogenous conditions (Figure 5E,F). This interaction was further supported by marked co‐localization of the two proteins, as visualized through co‐immunofluorescence (Figure 5G). Similarly, a direct interaction between PrPC and LYN was established using both Co‐IP and co‐immunofluorescence assays (Figure 5H–J). Using iterative IP‐re‐IP experiments, we demonstrated that PrPC simultaneously engages LYN and STAT3, facilitating the assembly of a novel ternary complex (Figure 5K). To further identify the key structural domains mediating the interaction between STAT3, LYN, and PrPC, we constructed plasmids expressing different truncated mutants of Flag‐tagged STAT3 and His‐tagged LYN (Figure 5L,M). Domain analysis revealed that PrPC directly binds to the N‐terminal domain (NTD) of STAT3 and the Src homology (SH) domain of LYN, respectively (Figure 5N,O).

To elucidate the role of PrPC in regulating the interaction between LYN and STAT3, we examined how PrPC expression modulates their association. Knocking down PRNP attenuated the LYN–STAT3 interaction, whereas overexpressing PRNP strengthened it, suggesting that PrPC expression positively correlates with the stability of the LYN–STAT3 complex (Figure 5P). Consistent with this, LYN knockdown in PRNP‐overexpressing cells substantially reduced STAT3 phosphorylation (Figure S10B). Nuclear‐cytoplasmic fractionation further revealed that LYN depletion markedly decreased nuclear p‐STAT3 levels, indicating that LYN regulates both STAT3 phosphorylation and its nuclear translocation (Figure S10C,D). Moreover, in PRNP‐overexpressing cancer cells, LYN knockdown strongly suppressed STAT3 phosphorylation and consequently inhibited STAT3‐driven cell migration and invasion (Figure S10E,F). In summary, our results demonstrate that PrPC acts as a critical scaffolding molecule that directly binds the NTD domain of STAT3 and the SH domain of LYN, thereby facilitating and stabilizing the LYN–STAT3 interaction and ultimately promoting STAT3 activation.

### 2.7 USP18 Maintained PrPC Stability Through Deubiquitination

**FIGURE 6:**
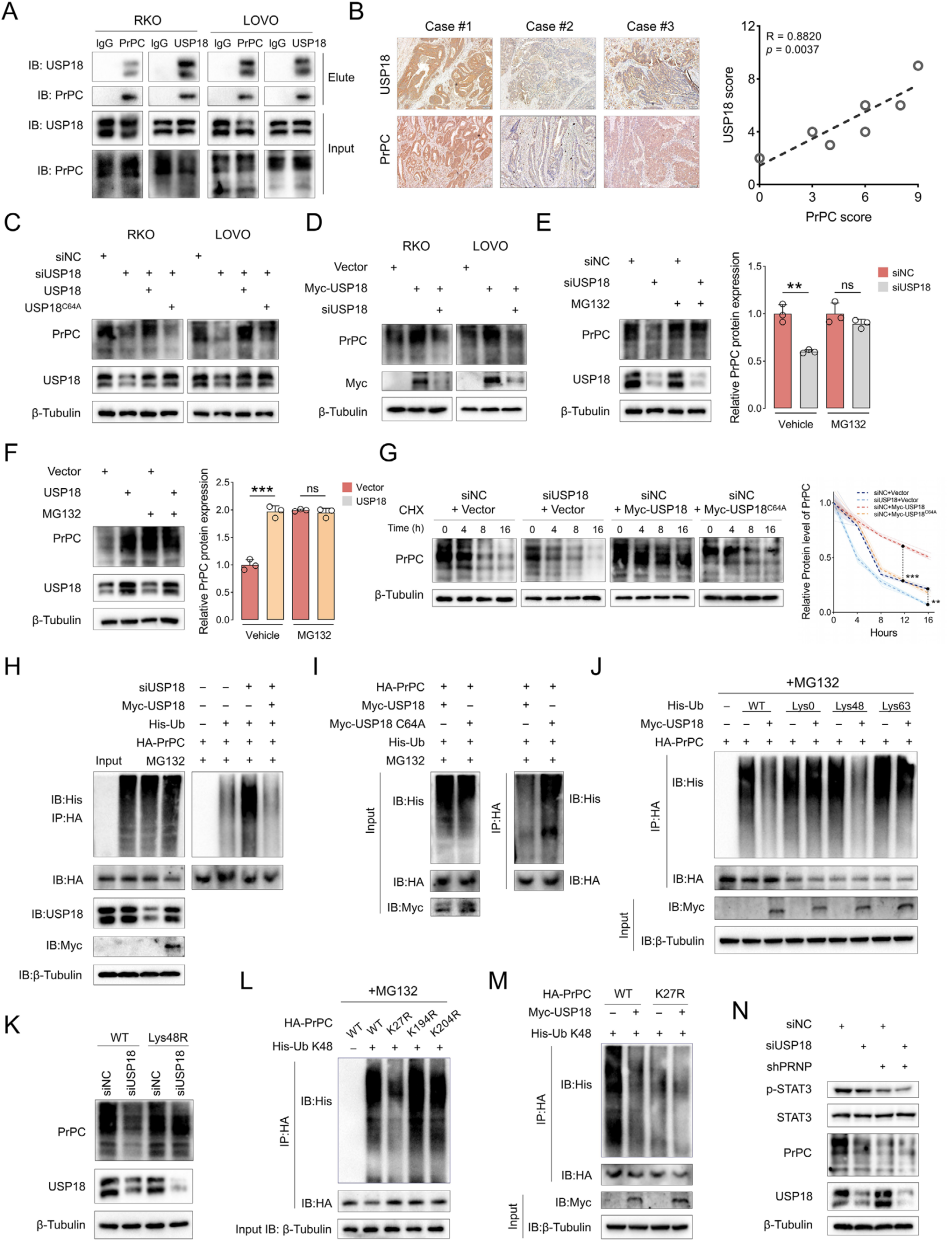
USP18 stabilizes PrPC to promote STAT3 phosphorylation in CRC cells. (A) Endogenous interaction between USP18 and PrPC in RKO and LOVO cells, analyzed by Co‐IP with the indicated antibodies, followed by western blot. IgG served as an isotype control. (B) Representative immunohistochemistry (IHC) images and correlation analysis of PrPC and USP18 expression in CRC tissues. (C) Western blot of PrPC levels in RKO cells expressing USP18 wild‐type (WT) or the catalytically inactive C64A mutant, with or without endogenous USP18 knockdown. β‐tubulin was used as a loading control. (D) Western blot of PrPC in RKO cells co‐transfected with Myc‐USP18 and siRNA targeting USP18, individually or in combination. (E) Western blot of PrPC in USP18‐silenced RKO cells treated with or without the proteasome inhibitor MG132. (F) Western blot of PrPC in USP18‐overexpressing RKO cells treated with or without MG132. (G) Determination of PrPC protein half‐life. RKO cells expressing siUSP18, Myc‐USP18, or Myc‐USP18 C64A were treated with cycloheximide (CHX) and harvested at the indicated time points for western blot analysis. (H) Analysis of total PrPC ubiquitination. RKO cells were co‐transfected with the indicated constructs (siUSP18, Myc‐USP18, and His‐Ub), treated with MG132, and analyzed by western blot. (I) Co‐IP analysis of PrPC ubiquitination in the presence of Myc‐USP18 WT or the C64A mutant. (J) Co‐IP analysis of PrPC ubiquitination in RKO cells co‐expressing Myc‐USP18, HA‐PrPC, and His‐Ub (WT, Lys0, Lys48 alone, or Lys63 alone). (K) Western blot of whole‐cell lysates from RKO cells transfected with siNC or siUSP18, along with Ub WT or the Lys48R mutant. (L) Effects of PrPC lysine‐to‐arginine mutations on its K48‐linked polyubiquitination. HA‐tagged PrPC WT or mutants were expressed in RKO cells along with Ub (K48 alone). (M) Quantification of the effects of USP18 on the K48‐linked polyubiquitination of WT and mutant PrPC. (N) Western blot analysis of p‐STAT3 (Tyr705) levels in RKO cells following genetic perturbations. Data are representative of at least three independent experiments (mean ± SD). ^**^ *p* < 0.01, ^***^ *p* < 0.001.

Having established the critical role of PrPC in CRC, we focused on identifying the upstream pathways that modulate PrPC to guide therapeutic development. We hypothesized that a deubiquitinase might be recruited upstream of PrPC to stabilize its expression. Screening of the IP/MS library identified the deubiquitinase USP18 as a potential interacting partner of PrPC. Given the previously reported oncogenic roles of USP18 in multiple cancers, it was selected as a putative direct upstream regulator of PrPC [30]. The interaction between PrPC and USP18 was confirmed using Co‐IP assays and protein–protein docking analysis in CRC cells (Figure 6A and Figure S11A). Co‐immunofluorescence assays demonstrated clear co‐localization between USP18 and PrPC in RKO and LOVO cells (Figure S11B). Moreover, we performed IHC staining of CRC tissue samples to further study the relationship between PrPC and USP18 expression. Notably, the results revealed a statistically significant correlation between PrPC and USP18 protein expression levels in CRC tissues (R = 0.8820, *p* = 0.0037) (Figure 6B). Transcriptomic analysis of GEO datasets revealed no correlation between the RNA levels of PRNP and USP18 (Figure S11C). Consistent with this observation, neither USP18 overexpression nor knockdown in RKO and LOVO cells altered PRNP mRNA levels (Figure S11D). Collectively, these findings support the potential post‐translational regulatory relationship between the two molecules.

We observed that USP18 knockdown markedly reduced PrPC expression, and this effect was rescued by co‐expression of wild‐type USP18, but not by the catalytically inactive C64A mutant (Figure 6C). Conversely, USP18 overexpression in RKO cells increased endogenous PrPC levels, which were reversed by co‐expression of USP18 siRNA (Figure 6D). To study the mechanism underlying USP18‐mediated regulation of PrPC degradation, we treated USP18‐depleted cells with the proteasome inhibitor, MG132. Notably, MG132 partially reversed the USP18 knockdown‐induced reduction in PrPC levels (Figure 6E). Similarly, MG132 attenuated the increase in PrPC induced by USP18 overexpression (Figure 6F). These results suggest that USP18 interacts with PrPC and inhibits its ubiquitin‐mediated proteasomal degradation, thereby promoting its stability. We then assessed the effect of USP18 expression on the stability of endogenous PrPC in the presence of the protein synthesis inhibitor, cycloheximide (CHX). USP18 depletion significantly accelerated PrPC degradation. Conversely, transfection with exogenous Myc‐USP18, but not Myc‐USP18 C64A mutant, retarded its degradation (Figure 6G). These findings demonstrate that USP18 directly regulates PrPC stability.

To further investigate whether USP18 regulates PrPC stability through the proteasomal degradation pathway, we performed a series of deubiquitination assays. RKO cells were co‐transfected with the indicated plasmids (siUSP18, Myc‐USP18, and His‐Ub) and treated with MG132 for polyubiquitination analysis. We verified that USP18 regulates the polyubiquitination status of PrPC in CRC cells, with USP18 suppression increasing polyubiquitination levels (Figure 6H). Conversely, reintroduction of Myc‐tagged USP18 into USP18‐deficient cells substantially reduced PrPC polyubiquitination. Subsequently, we co‐transfected RKO cells with either Myc‐USP18 or Myc‐USP18 C64A, together with HA‐PrPC, and detected PrPC ubiquitination levels. Notably, after MG132 treatment, the PrPC polyubiquitination level in the Myc‐USP18 C64A transfection group was significantly higher than that in the Myc‐USP18 group (Figure 6I), indicating that residue C64 is essential for the direct removal of ubiquitin chains from PrPC. We also examined the specificity of USP18 for the major ubiquitin linkage types. Ubiquitination assays revealed that USP18 preferentially removed Lys48‐linked ubiquitin chains from PrPC (Figure 6J). To validate the functional importance of K48‐linked polyubiquitination in USP18‐mediated regulation of PrPC stability, we expressed a lysine 48‐restricted ubiquitin mutant (Lys48R) in USP18‐knockdown RKO cells. Lys48R ubiquitin expression rescued the USP18 silencing‐induced decrease in PrPC levels (Figure 6K). To further identify the specific ubiquitination site targeted by USP18, we generated lysine‐to‐arginine mutations in PrPC. Subsequent ubiquitination analysis identified K27 as the critical residue through which USP18 mediated the PrPC deubiquitination (Figure 6L,M). Collectively, these data demonstrate that USP18 stabilizes PrPC by specifically removing Lys48‐linked polyubiquitin chains from K27 of PrPC.

Next, we studied whether USP18 regulates STAT3 pathway activation through PrPC. To determine whether USP18 requires PrPC to regulate p‐STAT3, we performed concurrent knockdown of both proteins. The results revealed that p‐STAT3 levels decreased upon PRNP knockdown alone; however, they remained stable in the double‐knockdown group (Figure 6N). Consistently, USP18 overexpressing partially rescued p‐STAT3 levels in PrPC‐deficient cells (Figure S11E). These data indicate that PrPC is indispensable for USP18‐mediated p‐STAT3 stabilization.

### 2.8 PrPC Demonstrated Potential as a Biomarker for CRC Screening

**FIGURE 7:**
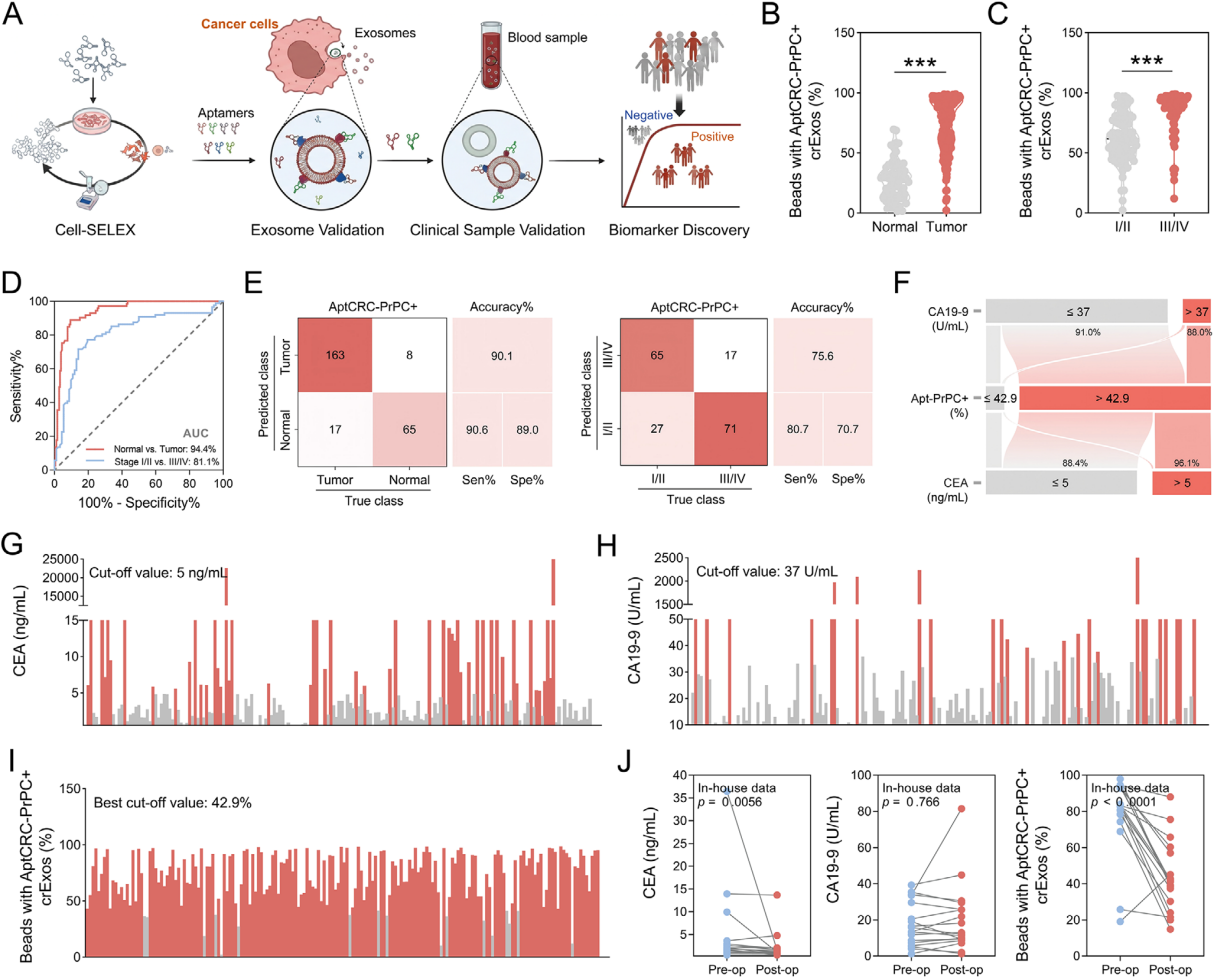
AptCRC‐PrPC+ circulating exosomes serve as a highly sensitive and specific liquid biopsy biomarker. (A) Schematic diagram of the discovery and validation of CRC‐derived exosome biomarkers using Cell‐SELEX technology. (B) Comparison of AptCRC–PrPC+ crExos levels between CRC patients (*n* = 180) and healthy controls (*n* = 73). (C) Comparison of AptCRC–PrPC+ crExos levels between patients with stage I/II (*n* = 92) and stage III/IV (*n* = 88). (D) Receiver operating characteristic (ROC) curves for AptCRC‐PrPC+ crExos in distinguishing normal vs. tumor (AUC = 94.4%) and stage I/II vs. III/IV CRC cases (AUC = 81.1%). (E) Confusion matrices of AptCRC‐PrPC+ crExos for normal vs. tumor and stage I/II vs. III/IV CRC classification, with overall accuracy, sensitivity (Sen%), and specificity (Spe%) metrics. (F) Alluvial diagram illustrating the patient distribution and diagnostic overlap among CA19‐9, CEA, and AptCRC‐PrPC+ crExos in CRC patients. Patients are stratified into positive or negative cohorts based on established clinical cut‐off values (37 U/mL for CA19‐9, 5 ng/mL for CEA) and the optimal diagnostic threshold (> 42.9%) for AptCRC‐PrPC+ crExos. (G–I) Distribution of patients by biomarker results (CEA (G), CA19‐9 (H), AptCRC‐PrPC+ crExos (I)). (J) Longitudinal changes in serum levels of CEA, CA19‐9, and AptCRC‐PrPC+ crExos in CRC patients before and after tumor resection.

Based on the aforementioned high binding affinity of WHY‐3E for tumor cell‐derived exosomes, we further investigated its diagnostic value for circulating exosomes (crExos) from patients with CRC to evaluate its clinical application potential as a liquid biopsy biomarker (Figure 7A). Subsequently, we designated the aptamer WHY‐3E targeting the PrPC protein as AptCRC‐PrPC. Subsequently, we isolated crExos from blood samples collected from patients with CRC (*n* = 180) and healthy donors (*n* = 73). Box plot statistics further confirmed that the level of AptCRC‐PrPC+ crExos was significantly elevated in the CRC group compared to the healthy controls, with a diagnostic accuracy of 90.1% and an AUC of 0.944 (Figure 7B,D,E). Additionally, we observed that AptCRC‐PrPC+ crExos were significantly elevated in advanced tumor stages, achieving an accuracy of 75.6% in distinguishing early‐stage from late‐stage disease (AUC = 0.811) (Figure 7C,D,E). Further comparison with conventional serum tumor biomarkers, CEA and CA19‐9, revealed that the detection rate of AptCRC‐PrPC+ crExos was 90.5%, substantially higher than the positive rates of CEA (28.3%) and CA19‐9 (13.9%) (Figure 7F–I), consistent with previous reports [31, 32]. Notably, in the subgroup of patients who were negative for these conventional tumor markers, the proportion of AptCRC‐PrPC+ was approximately 90% (Figure 7F). Together, these findings demonstrate that AptCRC‐PrPC+ crExos exhibit high diagnostic accuracy for CRC, with superior detection performance compared to CEA and CA19‐9.

Based on these findings, we aimed to assess the potential of AptCRC‐PrPC+ crExos for monitoring treatment progression in CRC. The analysis of paired samples collected preoperatively and 7 days postoperatively from 18 patients revealed a significant decrease in AptCRC‐PrPC+ crExos in 17 cases after surgery (Figure 7J). Conversely, CEA and CA19‐9 levels revealed no notable changes in most patients. Only three patients had elevated CEA levels preoperatively, two of whom exhibited a decrease postoperatively; only one patient had elevated CA19‐9 preoperatively, with a none‐significant reduction observed after surgery. These findings indicate that AptCRC‐PrPC+ crExos outperform traditional serum markers in auxiliary diagnosis and postoperative monitoring, exhibiting a high potential for clinical translation. In summary, the WHY‐3E aptamer specifically recognizes PrPC+ exosomes derived from CRC and exhibits favorable diagnostic accuracy and sensitivity to postoperative changes.

## 3 Discussion

Conventional biomarker discovery relies on mass spectrometry‐based proteomics, which requires extensive data processing and validation. As a powerful alternative, we developed WHY‐3E, a molecular tool based on Cell‐SELEX technology, which directly identifies the PrPC protein overexpressed on CRC cells. The robust interaction between the WHY‐3E aptamer and its target underscores the significant potential of aptamer‐based strategies for streamlined target identification and biomarker development.

Consistent with previous studies, we confirmed that PrPC is significantly upregulated in CRC tissue and is associated with poor patient prognosis [27]. Functional assays demonstrated that PrPC enhances the migratory and invasive capabilities of CRC cells, suggesting its role as a critical oncogene. While transcriptional upregulation is a common mechanism of oncogene overexpression, our study revealed a previously unrecognized layer of post‐translational control. We identified USP18 as a key upstream regulator that stabilizes PrPC, which significantly enhances the protein stability of PrPC by removing Lys48‐linked ubiquitin chains. USP18 has been widely reported to play multifaceted roles in various malignancies, functioning as a deubiquitinase and an interferon signaling inhibitor that remodels the tumor microenvironment [33, 34, 35]. USP18‐induced stabilization leads to a global increase in PrPC, which both fuels intracellular oncogenic signaling and enriches its presence on secreted exosomes. This dual effect reconciles the diagnostic target with the underlying pathogenesis, establishing exosomal PrPC as a reliable ‘mirror’ of the cellular oncogenic state.

As a GPI‐anchored protein, PrPC has traditionally been implicated in functions such as signal transduction and stress protection [36, 37]. However, in this study, we characterized the subcellular localization of PrPC in CRC cells and confirmed its stable expression in the cytoplasm. This observation aligns with the hypothesis proposed by Richard et al., which suggests that membrane‐bound and cytoplasmic forms of PrPC represent an identical protein entity and may undergo dynamic interconversion through processes such as endocytosis – recycling and trafficking – maturation [14, 38]. Moreover, utilizing cell‐surface biotinylation and endocytosis inhibition, our study demonstrated that membrane‐bound PrPC is the essential source of the functional cytoplasmic pool. Based on this dynamic trafficking link, the highly specific aptamer probe against PrPC offers an essential tool for deeply interrogating its complex intracellular biological roles. Mechanistically, RNA‐seq identified Moesin (MSN) as a direct downstream transcriptional target of PrPC. MSN, a member of the Ezrin‐Radixin‐Moesin (ERM) protein family, is vital for cell motility, adhesion, and signaling by associating the actin cytoskeleton with the plasma membrane [39, 40]. This function is exemplified by its interactions with key regulators, including MT1‐MMP and the E‐cadherin/p120‐catenin adhesion complex [41]. Given the established role of PrPC in regulating cell motility processes, including lamellipodia formation and EMT, we hypothesized that MSN may serve as a critical effector mediating the pro‐metastatic functions of PrPC [42]. Supporting this hypothesis, functional MSN knockdown markedly attenuated the malignant cancer cells phenotypes. These results collectively support the existence of a highly activated PrPC‐MSN signaling axis that drives CRC progression.

We have definitively established that Signal Transducer and Activator of Transcription 3 (STAT3) functions as a direct transcription factor for MSN. This discovery aligns with and mechanistically explains the established role of STAT3 in promoting tumor‐cell migration. STAT3 induces cytoskeletal remodeling and alters cell polarity by regulating the expression of key EMT effectors, such as E‐cadherin, Snail, and Vimentin [43, 44]. The function of its downstream target, MSN, is entirely consistent with this pro‐migratory program, positioning MSN as a critical executor of STAT3‐driven invasion. Furthermore, our study elucidated the precise molecular mechanism underlying LYN‐mediated STAT3 activation. Although the existence of the LYN‐STAT3 signaling axis has been documented, the regulatory context required for its specific activation remains unclear [45, 46]. Here, we demonstrate that this phosphorylation event is strictly dependent on cellular PrPC. PrPC acts as an indispensable molecular scaffold, simultaneously interacting with the N‐terminal domain (NTD) of STAT3 and the Src homology (SH) domain of LYN, to form a highly efficient signaling unit. This finding resonates with previous observations of the scaffolding potential of PrPC, exemplified by its cooperation with EGFR, and significantly expands this concept [11]. Our findings elevate PrPC from a passive signal relay at the plasma membrane to a master organizer of specific transcriptional complexes in the cytoplasm.

From a translational perspective, the PrPC‐LYN‐STAT3 complex emerges as a novel biomarker for predicting tumor aggressiveness and metastatic potential. Traditional kinase inhibitors are often plagued by toxicity and acquired resistance [47, 48]. Conversely, targeting the protein–protein interaction interface of PrPC offers a promising alternative strategy. By specifically disrupting the assembly of this oncogenic complex without globally inhibiting kinase activity, this approach could yield a highly specific and potent therapy to suppress cancer metastasis, potentially accompanied by a more favorable therapeutic window.

Liquid biopsy, particularly through peripheral blood analysis, holds immense promise for non‐invasive cancer screening. Tumor‐derived exosomes, which carry a rich cargo of proteins and nucleic acids, are a key focus in this area [49]. Despite this promise, a critical bottleneck remains: The lack of robust tools for the clinically specific identification of tumor‐derived exosomes [50]. To address this issue, based on the stabilization mechanism of PrPC and its dynamic distribution between intracellular compartments and exosomes, we developed a detection system using the WHY‐3E aptamer targeting crExos in CRC. This system demonstrated superior diagnostic efficacy and detection stability compared to traditional serum tumor markers. Furthermore, this aptamer‐based platform is non‐invasive, cost‐effective, and clinically accessible, presenting a novel strategy for CRC screening that has the potential to overcome the limitations of current paradigms.

However, this study has several limitations. First, USP18 mediates PrPC deubiquitination, thereby stabilizing its expression. However, the E3 ubiquitin ligase responsible for PrPC ubiquitination remains to be studied thoroughly. Second, although the pro‐cancer role of MSN has been well established, the underlying molecular mechanisms by which it promotes cancer metastasis in this pathway (e.g., whether it involves other cofactors or signaling pathways) remain to be further elucidated. Furthermore, the newly identified nucleic acid aptamer exhibits patient‐specific characteristics, and its diagnostic and monitoring utility will be validated in large‐scale clinical cohorts across different cancer types to facilitate its future application in non‐invasive diagnosis and disease surveillance.

In conclusion, we demonstrated that PrPC displayed an oncogenic role in promoting cell metastasis by directly targeting MSN expression. Mechanistically, we revealed that PrPC formed a ternary complex with LYN and STAT3, leading to STAT3 cascade overactivation and thereby regulating MSN expression. Furthermore, we demonstrated that USP18 interacted with PrPC and prevented its degradation in CRC cells. Consequently, the PrPC/LYN/STAT3/MSN axis may be a novel targetable signaling pathway for CRC treatment. Our study validated the malignant adaptor role of PrPC and revealed the therapeutic potential of targeting PrPC in CRC.

## 4 Materials and Methods

### 4.1 Clinical Tissue Specimens and Blood Sample Collection

Human CRC and adjacent non‐malignant tissues were procured from the Second Affiliated Hospital of Harbin Medical University. Histopathological confirmation of CRC or non‐malignant tissue status was performed by two independent pathologists to ensure accuracy and reliability.

Plasma samples were obtained from patients with CRC and healthy donors. All sample analyses were performed under blinded conditions with respect to clinical parameters and experimental groupings until the completion of the study. The tumor tissue and blood sample collection from patients with CRC was approved by the Ethics Committees of the Second Affiliated Hospital of Harbin Medical University and Zhejiang Cancer Hospital.

### 4.2 Cell Lines

The CRC cell lines used in this study were purchased from the Cell Bank of the Type Culture Collection Committee of Chinese Academy of Sciences (Shanghai, China) or Zhejiang Meisen Cell Technology Co., Ltd. All cell lines were cultured in a medium supplemented with 10% fetal bovine serum (FBS; GIBCO, Carlsbad, CA, USA) under a controlled environment of 5% CO_2_ at 37°C.

### 4.3 Aptamer Selection for CRC Cell Lines Using Cell‐SELEX

The aptamers were synthesized and purified by Sangon Biotech (Shanghai). Fluorescein isothiocyanate (FITC) and biotin were conjugated to the 5′ and 3′ ends, respectively. Lyophilized aptamers were reconstituted in D‐PBS, denatured at 95°C for 5 min, chilled on ice for 10 min, and equilibrated to room temperature before use. The prepared aptamers were stored at 4°C for short‐term use or −20°C for long‐term storage.

Cell‐SELEX was performed as previously described, with modifications [51]. A single‐stranded DNA (ssDNA) library containing a 45‐nt random core and fixed primer regions was incubated with HCT8 cells in binding buffer (D‐PBS with 4.5 g/L glucose, 1 mg/mL BSA, 0.1 mg/mL salmon sperm DNA, and 5 mm MgCl_2_) for 60 min at 4°C with shaking. Cells were washed to remove unbound sequences, scraped, and heat‐denatured. The recovered ssDNA was amplified using PCR using FAM‐labeled forward and biotin‐labeled reverse primers under 16–21 cycles, including a final extension at 72°C for 5 min.

FAM‐labeled ssDNA was isolated using streptavidin‐coated beads, desalted with an NAP‐5 column (Cytiva), vacuum‐dried, and used as the input for subsequent selection rounds. The washing stringency, reduced ssDNA input, and shorter incubation times were incrementally introduced over 12 rounds to enhance the specificity. The enriched library was cloned and sequenced by Sangon Biotech.

### 4.4 Aptamer Affinity Assay

The cells were dissociated with an enzyme‐free buffer, centrifuged, washed with D‐PBS, and resuspended in a binding buffer. Fluorescently labeled aptamers (200 nm) were two‐fold serially diluted across eight concentrations. Cell suspensions were incubated with diluted aptamers at 4°C for 30 min, washed, and resuspended in buffer for flow cytometry. A FAM‐labeled random‐sequence aptamer (Control sequence: TCGAGTTTACTGCACGCGGTATGACTGA) was used as a negative control to eliminate interference caused by non‐specific binding.

Data were analyzed using FlowJo software to assess the geometric mean fluorescence intensity for each concentration and blank control. Dissociation constants (K_d_) were derived by fitting the binding data to the equation Y = B_max_ × X / (K_d_ + X) via nonlinear regression using GraphPad Prism software (version 10.1). All experiments were performed in triplicate.

### 4.5 Exosomes Isolation

Exosomes from cell culture supernatants were isolated as described previously, with modifications [52]. Cells were grown in T225 flasks to 70%–80% confluence in medium containing 10% FBS and then switched to serum‐free medium for 48 h. The conditioned medium was collected, sequentially centrifuged at 800 × g for 5 min and 2000 × g for 30 min, filtered through a 0.22 µm membrane, and ultracentrifuged at 120 000 × g for 2 h at 4°C. The pellet was washed in PBS by repeated ultracentrifugation and finally resuspended in 100 µL PBS.

Plasma‐derived exosomes were isolated using the Invitrogen Total Exosome Isolation Kit. Cell‐free plasma (100 µL) was thawed on ice, mixed with 50 µL PBS and 30 µL reagent, incubated for 10 min at RT, and centrifuged at 10 000 × g for 5 min. The pellet was resuspended in 100 µL PBS for downstream applications.

### 4.6 Transmission Electron Microscope (TEM) of Exosomes

For TEM, exosome morphology was analyzed by applying 30 µL of suspension to carbon‐coated copper grids for 2–5 min. Excess liquid was blotted, and the grids were air‐dried for 10 min, stained with 2% uranyl acetate for 90 s, blotted, and dried for 3 h before imaging.

### 4.7 Flow Cytometry Analysis for Cells and Exosomes

For cell‐binding analysis, the cells were dissociated with enzyme‐free buffer, washed, and resuspended in D‐PBS. After centrifugation, the pellets were incubated with 200 nm FITC‐labeled aptamers in the binding buffer for 30 min at 4°C with gentle mixing. The cells were then washed twice with D‐PBS and resuspended in the washing buffer. An FITC‐labeled random‐sequence aptamer was used as a negative control. The aptamer‐positive cell percentage was determined by flow cytometry.

For gene knockdown validation experiments and aptamer binding assays with gene‐edited cells, HCT8‐shNC and shPRNP cells in good growth condition were incubated with antibodies (5 µg/mL) and aptamers (200 nm), respectively. Three parallel replicates were set for each group, resulting in a total of 12 experimental samples. For antibody staining, the primary antibody was incubated with the cells on ice in binding buffer for 30 min. After incubation, the cells were washed, followed by further incubation with the secondary antibody for 30 min. For aptamer staining, the aptamer WHY‐3E was directly incubated with the cells on ice in binding buffer for 30 min. After aptamer incubation, the cells were washed twice and resuspended in 200 µL of washing buffer. After all samples were processed, flow cytometry was performed.

For exosome binding analysis, 30 µg of exosomes were coupled to aldehyde/sulfate latex beads (4 µm, Invitrogen) for 15 min at room temperature, followed by incubation in PBS for 30 min. The reaction was quenched using 100 mm glycine and 2% BSA. After centrifugation, beads were blocked with 10% BSA and incubated with 200 nm fluorescently labeled aptamers for 30 min. The beads were washed and resuspended in the washing buffer. The mutant aptamer and FITC‐labeled anti‐CD63 antibody (SinoBiological) were used as negative and positive controls, respectively. The percentage of positive beads was quantified using flow cytometry. Flow cytometry was performed on a Beckman Coulter instrument, and data were analyzed using FlowJo software (version 10.8.1).

### 4.8 Surface Plasmon Resonance (SPR) Experiments

SPR was performed on a Biacore‐8K instrument (Cytiva) at 25°C. Ligands were immobilized on a CM5 sensor chip via standard amine‐coupling chemistry using a 7‐min activation with a mixture of 0.2 m EDC and 0.05 m NHS. Ligands, diluted to 50 µg/mL in 10 mm sodium acetate (pH 4.5), were injected at 10 µL/min for 10 min. The remaining active groups were blocked with 1 m ethanolamine (pH 8.5).

Aptamers were serially diluted from 3.1 to 200 nm in running buffer (10 mm HEPES, 150 mm NaCl, 0.005% polysorbate 20, pH 4.5) and injected over the sensor surface at 30 µL/min. The association and dissociation were monitored for 200 s each. Kinetic parameters were derived from the sensorgrams using the Biacore‐8K evaluation software with a 1:1 binding model.

Binding data and K_d_ values were determined using Biacore‐8K software. The sensorgrams were fitted to a 1:1 binding model to derive the kinetic parameters, as previously described [53].

### 4.9 Target Protein Identification of Aptamer by Stable Isotope Labeling by Amino Acids in Cell Culture (SILAC)‐Based Quantitative Proteomics

The target proteins bound to the aptamer were identified using a modified SILAC approach. HCT8 cells were cultured for six generations in lysine/arginine‐deficient RPMI‐1640 medium supplemented with either “heavy” or “light” isotopic forms of these amino acids and 10% FBS. Biotinylated aptamers (100 nm) were denatured at 95°C, renatured on ice, and incubated with heavy‐ or light‐labeled cells (2 × 10^8^) in binding buffer at 4°C for 30 min. Crosslinking was performed using 2% formaldehyde (4°C, 15 min) and quenched with glycine. Cells were washed with PBS/EDTA and lysed in a buffer containing sodium dodecyl sulfate (SDS), Triton X‐100, EDTA, PMSF, and protease inhibitors. The lysates were incubated with streptavidin agarose beads (Cytiva) at 4°C for 1 h. The beads were washed sequentially with lysis buffer, washing buffer, and PBS. The bound proteins were eluted in ammonium bicarbonate and heated at 65°C overnight. Samples were boiled in SDS loading buffer, resolved by 12% SDS‐PAGE, and the target bands were excised for MS analysis.

Proteins were identified using an LTQ‐Orbitrap Velos mass spectrometer (Thermo Fisher Scientific). Raw data were processed with MaxQuant and searched against the UniProt database.

### 4.10 Cell Transfection

The short hairpin RNAs (shRNAs) used in this study were synthesized by Genomeditech (Shanghai, China). The small interfering RNAs (siRNAs) were synthesized by Genecefe Biotechnology (Jiangsu, China). Overexpression plasmids for PRNP, Flag‐STAT3, His‐LYN, Myc‐USP18, and His‐Ub were acquired from MiaoLingPlasmid (Wuhan, Hubei, China). Additional truncated or mutant plasmids were also obtained from Genomeditech (Shanghai, China). All shRNAs, siRNAs, and plasmids were transfected using Lipofectamine 3000 (Invitrogen). The sequences of shRNAs and siRNAs were listed in Table S1.

### 4.11 RNA Extraction and Quantitative PCR

Total RNA was extracted from the CRC cell lines using TRIzol reagent (Invitrogen). cDNA was synthesized from 2 µg RNA using the High‐Capacity cDNA Reverse Transcription Kit (TOYOBO). Quantitative PCR was performed using SYBR Green (Invitrogen) on an Applied Biosystems 7500 instrument, with 2 µg cDNA per reaction. Gene expression was quantified using the 2^−ΔΔCt^ method normalized to GAPDH. The primer sequences are provided in Table S2.

### 4.12 Western Blotting (WB) Assay

Total proteins were extracted and quantified using a BCA assay (APPLYGEN). Proteins were separated by 10% SDS‐PAGE and transferred to nitrocellulose membranes. After blocking with 5% non‐fat milk in TBST, the membranes were incubated with primary antibodies at 4°C overnight, followed by HRP‐conjugated secondary antibodies for 2 h at room temperature. Signals were detected using an ECL kit (APPLYGEN) and imaged using a Bio‐Rad ChemiDoc MP system. The antibodies used are listed in Table S3.

### 4.13 Immunohistochemistry (IHC) Staining

The tissue sections were dewaxed and rehydrated. Subsequently, heat‐induced antigen retrieval was performed. Endogenous peroxidases were quenched with hydrogen peroxide, and nonspecific binding was blocked using 5% BSA. Finally, the sections were incubated with primary antibodies overnight at 4°C, followed by incubation with the appropriate secondary antibodies. Detection was performed using DAB substrate, and the nuclei were counterstained with hematoxylin.

Immunohistochemical staining of the CRC tissue microarray was assessed independently by three observers, including at least one pathologist. The staining intensity was graded as 0 (negative), 1 (weak), 2 (moderate), or 3 (strong). The percentage of positive cells was scored as 1 (0%–25%), 2 (26%–50%), 3 (51%–75%), or 4 (76%–100%). The final IHC score for each sample was calculated by multiplying the intensity score by the positive cell percentage score.

### 4.14 Co‐Immunoprecipitation (Co‐IP) Assay

Protein–protein interactions were assessed by co‐immunoprecipitation using a commercial kit (Beyotime). The antibodies were first incubated with agarose beads and then washed thrice with TBS. The antibody‐bound beads were then incubated with the cell lysates to facilitate coupling. After three additional TBS washes, the complexes were eluted by boiling in 1× SDS loading buffer for 10 min. The immunoprecipitated proteins were analyzed using immunoblotting.

### 4.15 Immunofluorescence (IF) Assay

For subcellular localization analysis, CRC cells were incubated with 200 nm FITC‐labeled WHY‐3E at 4°C for 40 min. After washing, the cells were fixed with 4% paraformaldehyde, and the nuclei were counterstained with DAPI. Images were acquired using a confocal laser scanning microscope.

The cells grown on coverslips were washed with PBS and fixed with 4% paraformaldehyde for 20 min. After permeabilization with 0.2% Triton X‐100 for 15 min and washing with ice‐cold PBS, samples were blocked with 5% goat serum (Sangon Biotech) in PBST for 1 h at room temperature. Primary antibodies were applied overnight at 4°C, followed by incubation with Alexa Fluor 488‐conjugated goat anti‐mouse IgG or Alexa Fluor 594‐conjugated goat anti‐rabbit IgG for 1.5 h. The nuclei were counterstained with DAPI before imaging using fluorescence microscopy.

### 4.16 Wound Healing and Transwell Migration and Invasion Assay

Transfected cells were grown in 6‐well plates to until they reached 90% confluence. A scratch wound was introduced using a 1 mL pipette tip, and the cells were subsequently cultured in serum‐free medium. Migration was monitored by imaging at the indicated time points. Wound closure was quantified as the percentage of the area recovered relative to the initial scratch.

Cell migration and invasion were assessed using Transwell chambers (LABSELECT). For the invasion assays, the upper chamber was pre‐coated with Matrigel (Corning). The Cells (0.5‐2 × 10^5^) in serum‐free medium were seeded into the upper chamber, and the lower chamber contained 10% FBS as a chemoattractant. After 24–48 h, cells that migrated or invaded through the membrane were fixed with 4% formaldehyde, stained with 0.5% crystal violet, and quantified using the ImageJ software.

### 4.17 Cell Proliferation and Colony Formation Assays

Cell proliferation was evaluated using the Cell Counting Kit‐8 (CCK8; Yeasen). Briefly, transfected cells were seeded in 96‐well plates at 1000 cells per well and cultured for 24, 48, 72, and 96 h. Subsequently, CCK‐8 solution was added to each well, followed by a 2‐h incubation. The absorbance was measured at 450 nm to quantify cell viability.

For the colony formation assay, 1000 transfected cells were plated in 6‐well plates and cultured for two weeks. The resulting colonies were fixed with 4% paraformaldehyde, stained with 2% crystal violet, and then counted under a microscope after imaging.

### 4.18 Chromatin Immunoprecipitation (ChIP) Assay

ChIP assays were performed using a ChIP assay kit (Beyotime). Formaldehyde‐crosslinked cells were lysed and incubated with 2 µg of anti‐STAT3 antibody or normal IgG. DNA‐protein complexes were immunoprecipitated with magnetic beads, de‐crosslinked, and purified. The precipitated DNA was amplified by qPCR using promoter‐specific primers (Table S4).

### 4.19 Luciferase Reporter Assay

Transcriptional activity was assessed using a dual‐luciferase reporter assay. The WT or MUT promoter sequences were cloned into the pPro‐RB‐Report vector (RiboBio) and co‐transfected into CRC cells for 48 h. Luciferase activity was measured using the Dual‐Luciferase Assay Kit (Promega), with Renilla luciferase as the internal control. Reporter activation was expressed as the ratio of firefly to Renilla luciferase activity.

### 4.20 Subcellular Fractionation and Protein Extraction

To analyze the protein distribution across different compartments, subcellular fractions were prepared. Specifically, membrane and cytoplasmic proteins were isolated using a dedicated extraction kit (Epizyme), while nuclear and cytoplasmic proteins were obtained using the Beyotime Nuclear and Cytoplasmic Protein Extraction Kit (Beyotime). Both procedures were performed strictly according to the manufacturers' instructions.

### 4.21 Cleavable Cell‐Surface Biotinylation and Internalization Assay

Cell surface proteins of CRC cells were first labeled with Sulfo‐NHS‐SS‐Biotin (Thermo Fisher Scientific) at 4°C to arrest membrane trafficking. After quenching unreacted biotin, endocytosis was induced by incubating the cells in pre‐warmed complete medium at 37°C for specified durations (0, 15, 30, and 60 min). At each time point, membrane trafficking was rapidly halted by transferring the cells to ice. To strip the remaining biotin from uninternalized surface proteins, cells were treated with a cell‐impermeable MesNa‐based reducing buffer, followed by quenching with iodoacetamide (IAA). Subsequently, cells were lysed in RIPA buffer. Equivalent amounts of total cellular protein from each group were incubated with Streptavidin magnetic beads (Pierce) to capture the internalized biotinylated proteins, while a fraction of the lysate was retained as the input control. After stringent washing, the captured proteins were eluted by boiling in a reducing SDS loading buffer and analyzed by WB.

### 4.22 Reagents and Cell Treatments

The specific dynamin GTPase inhibitor Dynasore was purchased from (Beyotime) and dissolved in DMSO to prepare a stock solution. For endocytosis blockade experiments, CRC cells were washed with PBS and pre‐incubated with 50 µm Dynasore in serum‐free medium for 30 min at 37°C. Subsequently, the cells were subjected to specific assays while maintaining the presence of 50 µm Dynasore throughout the experimental period. Control cells were treated with an equal volume of DMSO under identical conditions.

### 4.23 Animal Experimental Procedures

All animal procedures were approved by the Institutional Animal Care and Use Committee. Six‐week‐old male athymic BALB/c nude mice were housed in specific pathogen‐free conditions. For intrasplenic injection, mice were anesthetized with pentobarbital sodium (1.5 mg/20 g, i.p.), and the spleen was exposed through a small flank incision. A suspension of 5 × 10^6^ RKO cells in 50 µL PBS was slowly injected using a 32 G needle. The injection site was gently compressed for 5 min to minimize leakage and facilitate tumor cell migration to the liver. After 6 weeks, the mice were euthanized, liver metastases were counted, and liver weights were measured for statistical comparison.

### 4.24 Bioinformatic Data

All samples used for clinical statistical analysis were retrieved from The Cancer Genome Atlas (TCGA; https://www.genome.gov/Funded Programs Projects/Cancer‐Genome‐Atlas) and Gene Expression Omnibus (GEO; https://www.ncbi.nlm.nih.gov/geo/) databases (GSE41568, GSE4183, GSE20916, GSE39582, GSE109057, and GSE40967) using a standardized processing pipeline.

### 4.25 Statistical Analysis

All statistical analyses were performed using GraphPad Prism software (version 9.0; La Jolla, CA, USA) and the Statistical Package for Social Sciences (version 22.0; Chicago, IL, USA). Data are presented as the mean ± standard deviation. Group differences were determined using Student's t‐test or one‐way analysis of variance, as appropriate. Survival rates were analyzed using Kaplan–Meier curves and compared using the log‐rank test. Time‐dependent changes in the internalization ratio of the target protein were analyzed separately. The overall effect of time (0–60 min) was assessed using repeated‐measures ANOVA, with Greenhouse‐Geisser correction applied if the sphericity assumption was violated. Linear trend analysis via simple linear regression was then performed. All experiments were independently repeated at least thrice to ensure reproducibility. A *p*‐value less than 0.05 was considered statistically significant (^*^ *p* < 0.05, ^**^ *p* < 0.01, ^***^ *p* < 0.001, and ^****^ *p* < 0.0001).

## Author Contributions

C.W., H.W., and C.Z. contributed equally to this study. C.W., T.B., H.H., M.W., and G.W. contributed to conceptualization. C.W., H.W., C.Z., H.Z., T.B., H.H., and G.W. contributed to methodology. C.W., C.Z., Y.L., and H.W. contributed to the software. Y.L., H.W., C.Z., Y.C., Y.Z., H.J., C.W., H.Z., X.W., and Z.C. contributed to validation. C.W., X.W., H.W., and J.W. contributed to formal analysis. C.Z., W.Z., Y.L., J.W., Z.C., Y.C., M.W., T.B., Y.Z., and G.W. contributed to the investigation. M.W., H.W., C.Z., Z.C., J.X., H.H., and T.B. contributed to resources. Y.W., J.L., H.W., and N.Z. contributed to data curation. C.W., X.W., and H.W. contributed to the writing of the original draft. C.W., C.Z., M.W., T.B., H.H., and G.W. contributed to writing, review, and editing. H.W., Y.W., H.J., C.Z., and J.X. contributed to visualization. C.Z., J.L., N.Z., W.T., and G.W. contributed to supervision. C.W., W.Z., Z.Y., and C.Z. contributed to project administration. G.W., M.W., T.B., H.H and H.Z. contributed to funding acquisition.

## Ethics Statement

All specimens and relevant clinical information involved in this study were approved by the Medical Ethics Committee of the Second Affiliated Hospital of Harbin Medical University and Zhejiang Cancer Hospital (Ethics No.: YJSDW2024‐247; IRB‐2023‐1182(IIT)). Written informed consent was obtained from all participants. This study was carried out in strict accordance with the ethical principles of the Declaration of Helsinki. All animal experiments were performed under the approval of the Ethics Committee of the Second Affiliated Hospital of Harbin Medical University (Ethics No.: YJSDW2024‐247).

## Conflicts of Interest

The authors declare no conflicts of interest.

## Supporting information




**Supporting File**: advs75758‐sup‐0001‐SuppMat.doc.

## Data Availability

The data that support the findings of this study are available from the corresponding author upon reasonable request.
