# Supplementary material for: Aptamer‐Targeted PrPC Drives Colorectal Cancer Metastasis via a LYN‐STAT3 Complex and Enables Liquid Biopsy Detection
Source: Adv Sci (Weinh). 2026 May 19:e75758. Online ahead of print. doi: 10.1002/advs.75758 (PMC13335878; doi:10.1002/advs.75758)
Supplement: Supplementary file 1 — Supporting File: advs75758‐sup‐0001‐SuppMat.doc. [file ADVS-9999-e75758-s001.doc]

**Supporting Information**

**Aptamer-Targeted PrPC Drives Colorectal Cancer Metastasis via a LYN-STAT3 Complex and Enables Liquid Biopsy Detection**

*Chunlin Wang^1,#^, Hongyu Wu^2,3,#^, Chaojing Zheng^2,4,#^, Hao Zhang^1^, Xiaoqiu Wu^2^, Jiaqi Wang^1^, Zewen Chang^5^, Jun Xiang^1^, Yunxiao Liu^1^, Chenkai Zhang^1^, Yuliuming Wang^1^, Hao Jiang^1^, Yuchen Zhong^6^, Jun Luo^7^, Ying Chen^8^, NaNa Zhang^1^, Weiyuan Zhang^1^, Ziming Yuan^1^, ChaoXia Zou^9^, Weihong Tan^2,3^, Meng Wang^2,7*^, Hanqing Hu^1,*^, Tao Bing^2,7*^, Guiyu Wang^1,*^*

#These authors equally contribute to this work

*Correspondence authors:

Professor Guiyu Wang, Department of Colorectal Cancer Surgery, The Second Affiliated Hospital of Harbin Medical University, Harbin, Heilongjiang 150086, P.R. China

E-mail: H05920@hrbmu.edu.cn

Professor Tao Bing, The Key Laboratory of Zhejiang Province for Aptamers and Theranostics, Zhejiang Cancer Hospital, Hangzhou Institute of Medicine (HIM), Chinese Academy of Sciences, Hangzhou, Zhejiang 310022, P.R. China

E-mail: bingtao@him.cas.cn

Professor Hanqing Hu, Department of Colorectal Cancer Surgery, The Second Affiliated Hospital of Harbin Medical University, Harbin, Heilongjiang 150086, P.R. China

E-mail: huhanqing@hrbmu.edu.cn

Professor Meng Wang, Department of Colorectal Cancer Surgery, Zhejiang Cancer Hospital, Hangzhou Institute of Medicine (HIM), Chinese Academy of Sciences, Hangzhou, Zhejiang 310022, P.R. China

E-mail: wangmeng@zjcc.org.cn

**File list**

Figure S1-11

Table S1-4


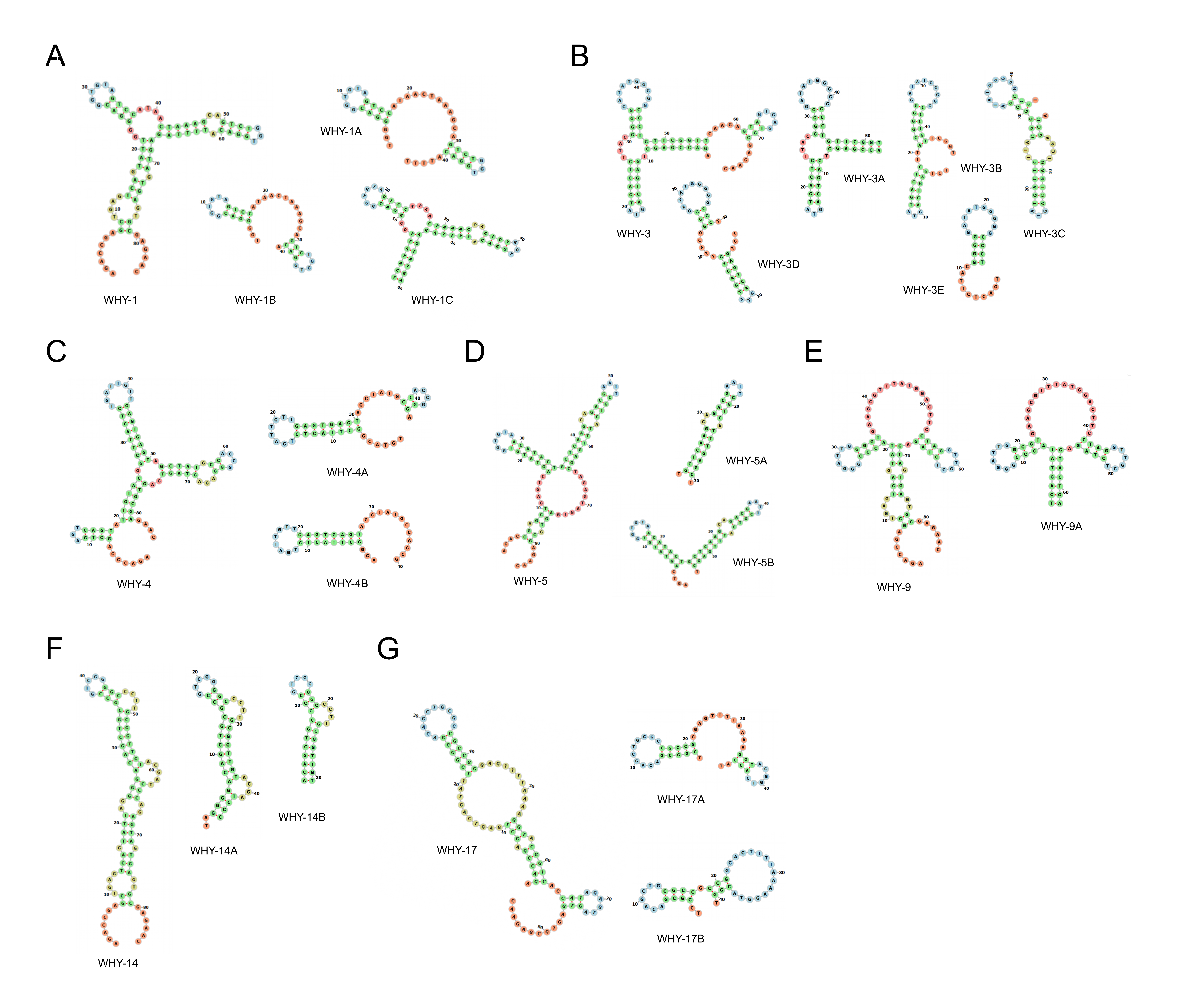


**Figure S1.** Predicted secondary structures of full-length aptamers and their truncated variants. The predicted secondary structures were performed for relatively high-abundance aptamers using UNAFold, followed by truncation into various forms guided by empirical knowledge.


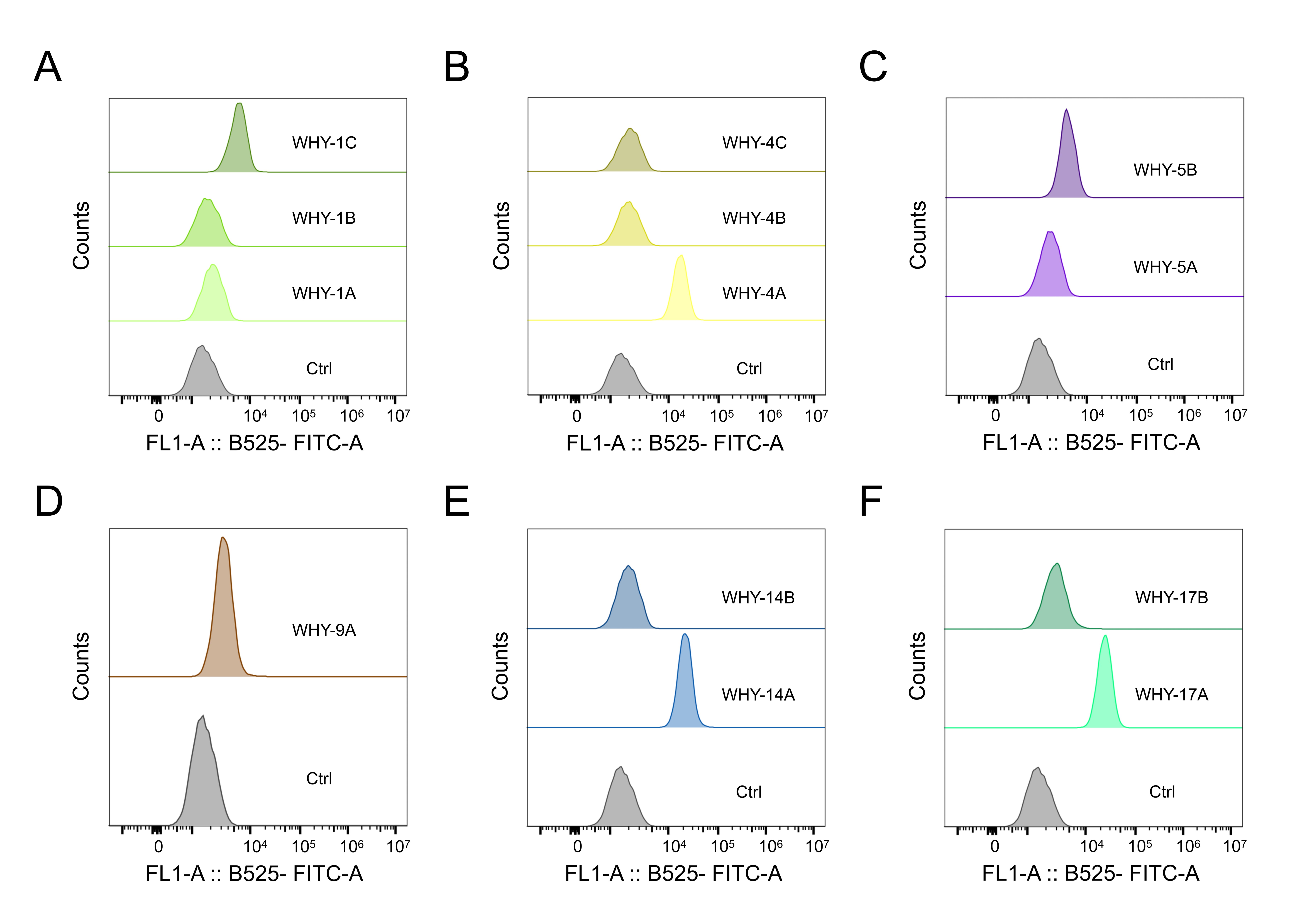


**Figure S2.** Flow cytometry results showing the binding ability of optimized aptamers to CRC cell line.


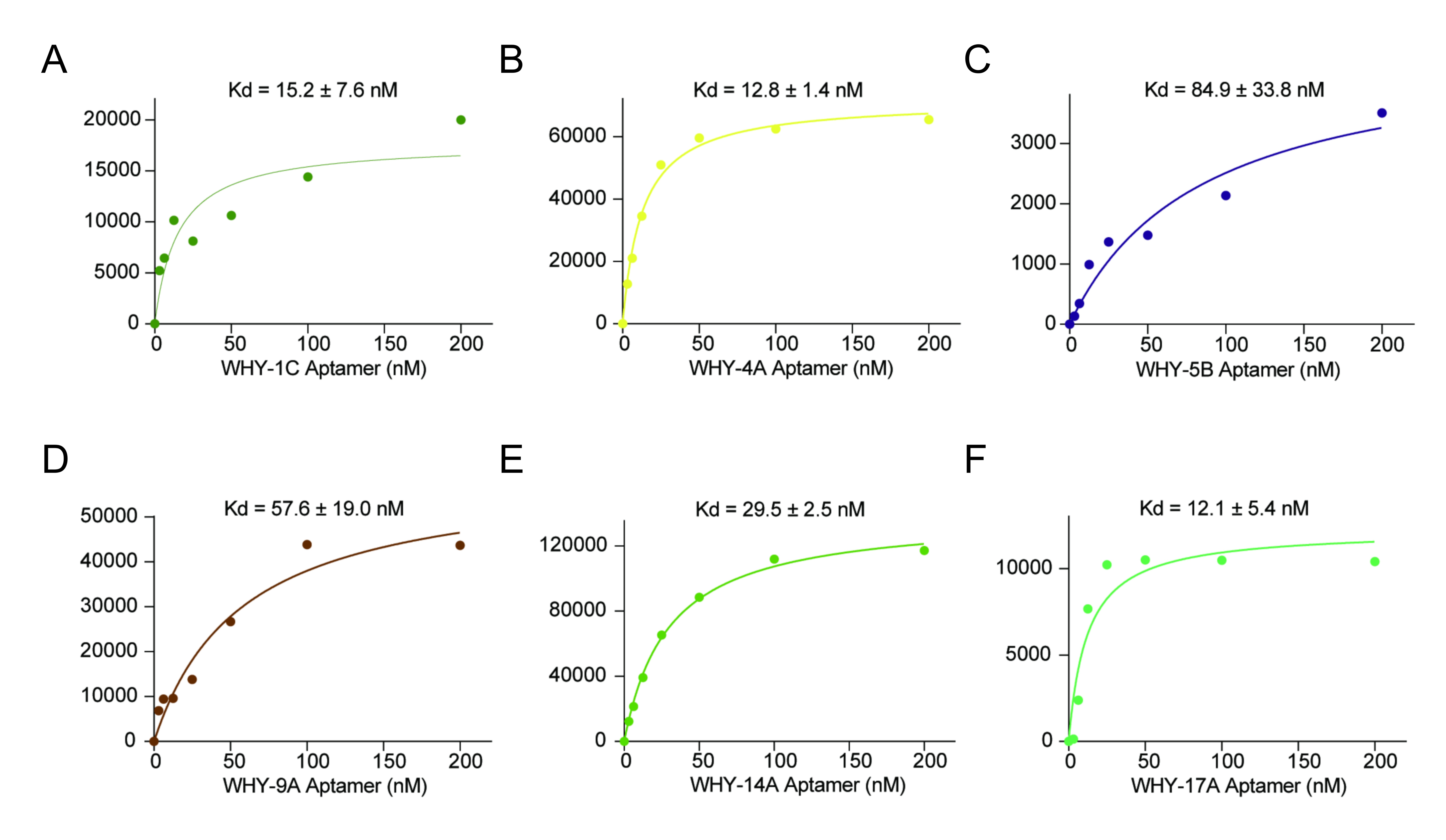


**Figure S3.** The dissociation constants (Kd) for each aptamer toward CRC cell lines assessed by flow cytometry. (A) WHY-1C, 15.2 ± 7.6 nM; (B) WHY-4A, 12.8 ± 1.4 nM; (D) WHY-5B, 84.9 ± 33.8 nM; (E) WHY-9A, 57.6 ± 19.0 nM; (F) WHY-14A, 29.5 ± 2.5 nM; and (G) WHY-17A, 12.1 ± 5.4 nM. The saturation binding curves illustrate the relationship between aptamer concentration and the extent of binding, demonstrating the distinct affinity profiles of the aptamers for target cells.


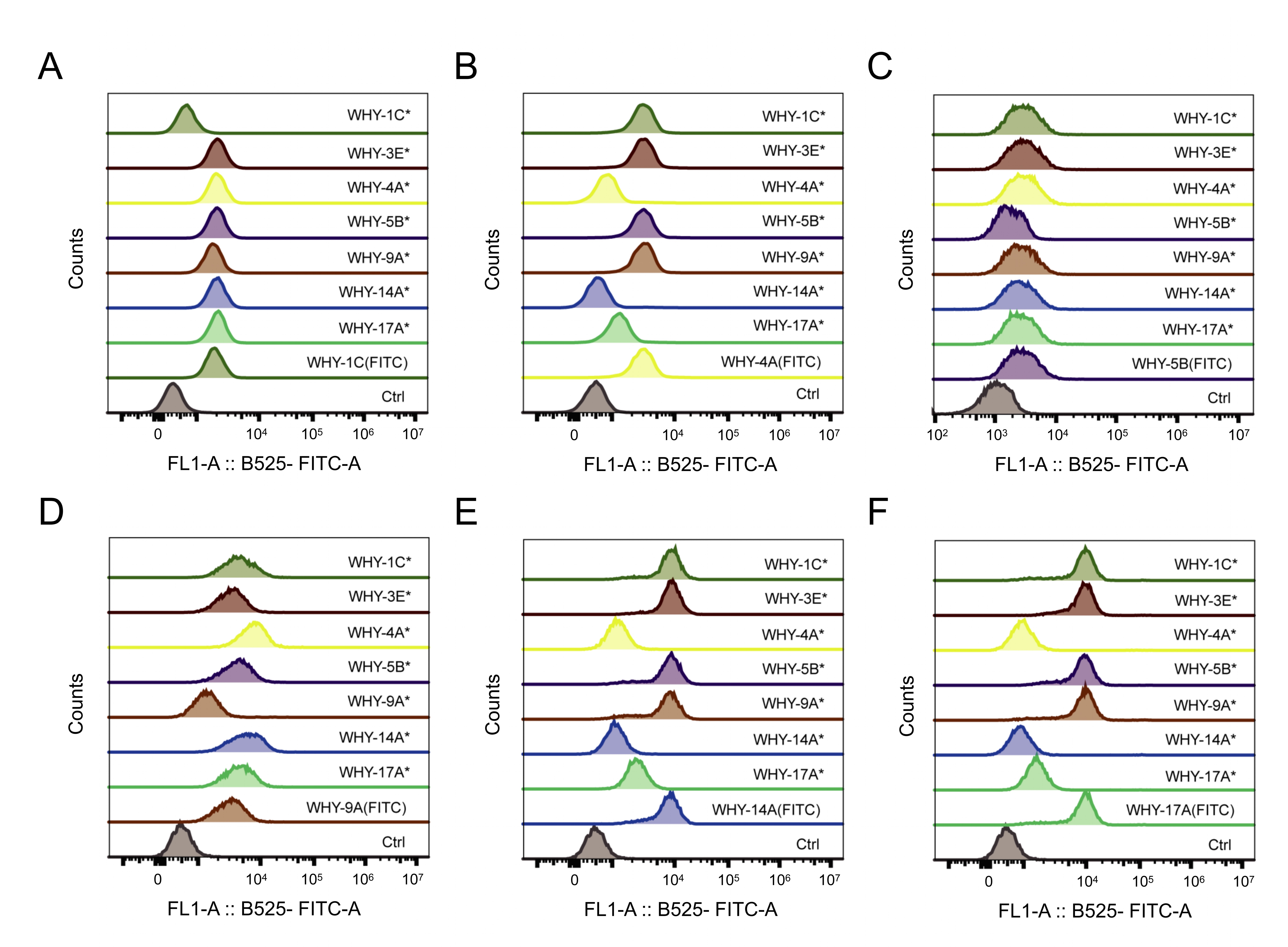


**Figure S4.** Competitive binding analysis among different aptamers assessed by flow cytometry. Unlabeled aptamers were co-incubated with their FITC-labeled counterparts, and fluorescence intensity was measured. A decrease in fluorescence indicates competition between the two aptamers for the same binding site, suggesting recognition of overlapping epitopes. Panels A-F show fluorescence signals for different aptamer pairs. FAM-conjugated aptamers are denoted as FITC-labeled; the asterisk (*) indicates unlabeled, unmodified aptamers.


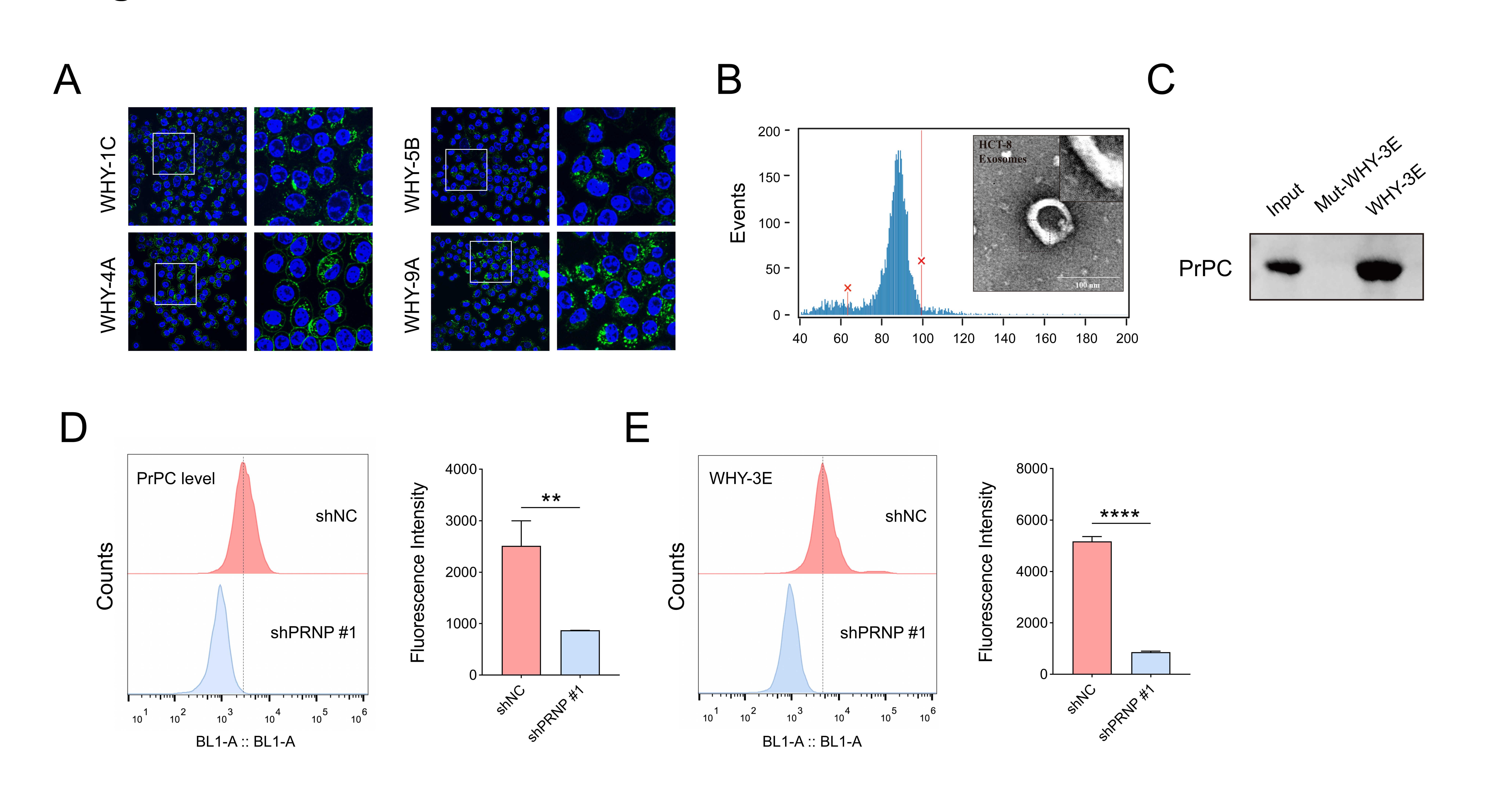


**Figure S5.** Screening for CRC-targeting aptamers and identifying the target of aptamer WHY-3E using Cell-SELEX.

(A) Immunofluorescence staining showing the subcellular binding morphology of FITC-labeled candidate aptamers (green) in HCT8 cells, with nuclei stained by DAPI (blue). (B) Characterization of CRC cell-derived exosomes: nanoparticle tracking analysis (NTA) for size distribution and transmission electron microscopy (TEM) for morphology. (C) Aptamer-mediated pull-down assay confirming the specific binding of WHY-3E to PrPC. Samples were stained with a PrPC antibody. (D) Flow cytometry analysis validating the PRNP knockdown efficiency in HCT8 cells, with representative histograms shown on the left and the corresponding quantification of the mean fluorescence intensity on the right. (E) Flow cytometry analysis evaluating the binding capacity of the fluorescently labeled WHY-3E to the cells, with representative histograms shown on the left and the corresponding quantification of the mean fluorescence intensity on the right.

**

Figure S6.** PrPC is overexpressed in CRC and correlates with poor clinical outcomes.

(A-B) Western blot analysis of PrPC protein levels in 8 pairs of human CRC (T) and matched adjacent non-tumor (N) tissues. β-tubulin was used as a loading control. (B) shows the quantitative analysis of the blots presented in (A). (C-E) Representative immunohistochemistry (IHC) images (C) and quantitative analysis (D-E) demonstrating upregulated PrPC expression in primary CRC and matched liver metastatic tissues from Cohort I. (F-G) IHC analysis of PrPC expression (G) and quantitative analysis (H) in a CRC tissue microarray (Cohort II). (H) COX analysis for overall survival (OS) using data from GSE40967. (I-J) Kaplan-Meier survival curves of OS in CRC patients from the GSE40967 (I) and TCGA (J) datasets, stratified by high vs. low PRNP expression.


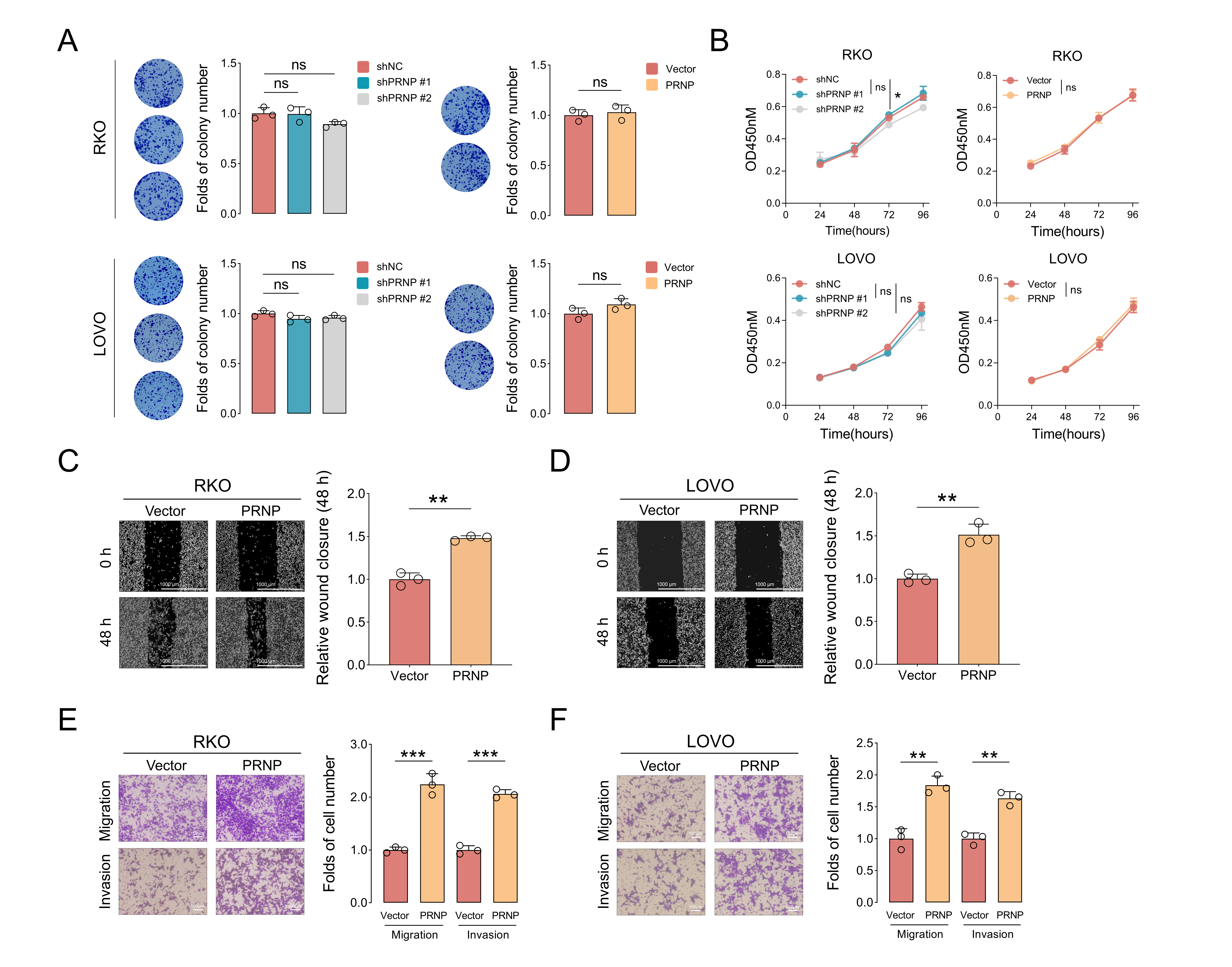


**Figure S7.** PrPC promotes metastasis of CRC in vitro and in vivo.

(A) Colony formation assays showing the promotive effect of PRNP overexpression on the proliferative capacity of RKO and LOVO cells. Representative images (left) and quantitative analysis (right) are presented. (B) Cell Counting Kit-8 (CCK-8) assays assessing the effect of PRNP overexpression or knockdown on the viability of RKO and LOVO cells. (C-D) Transwell assays evaluating the effect of PRNP overexpression on cell migration. Representative images at 0 and 48 hours (left) and quantitative analysis of wound closure rates (right) are presented. (E-F) Wound healing assays demonstrating the effect of PRNP overexpression on cell migration and invasion. Representative images (left) and corresponding quantification (right) are shown.


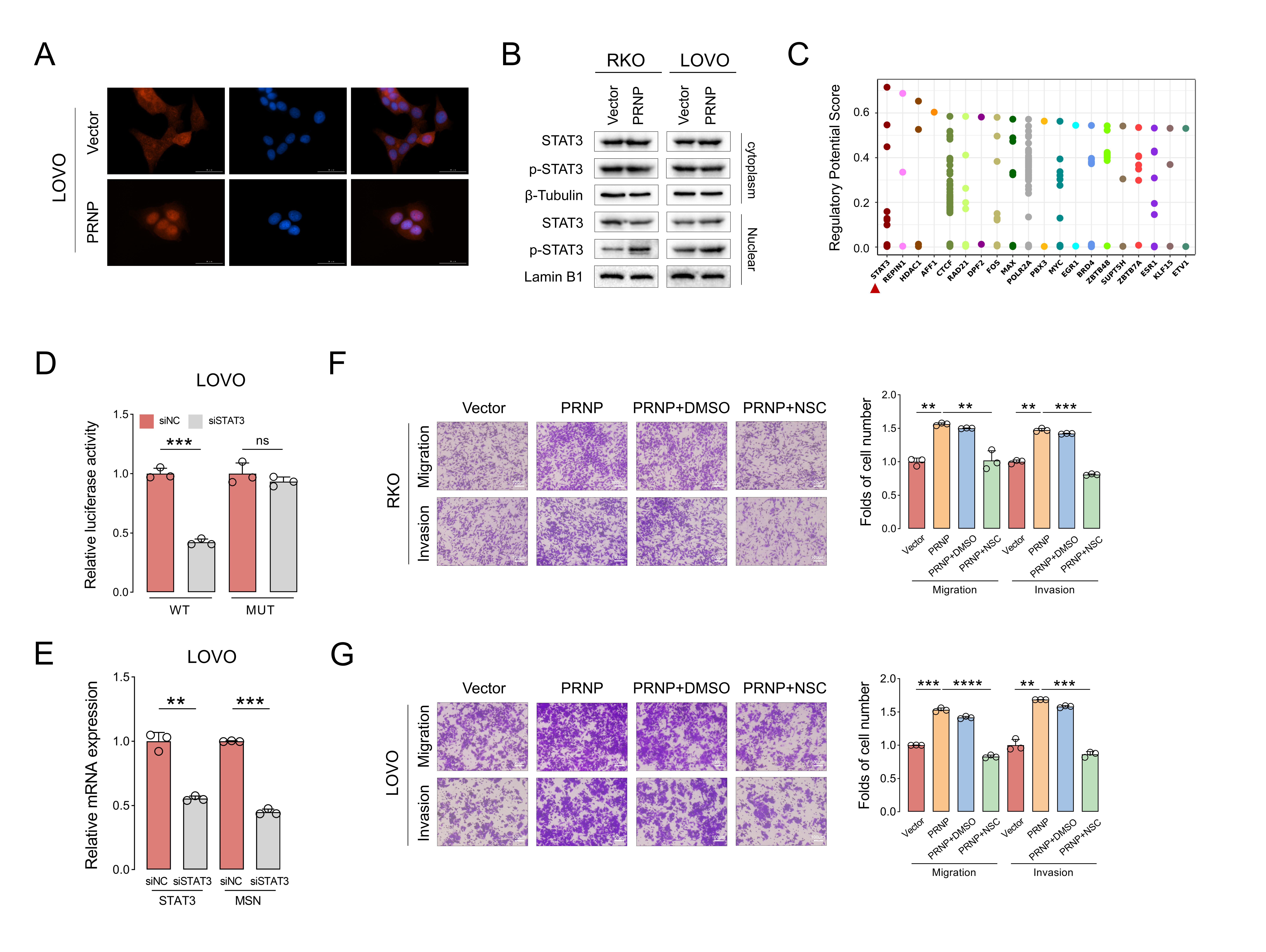


**Figure S8.** PrPC regulates MSN expression via STAT3 phosphorylation.

(A) Immunofluorescence staining showing PrPC-induced nuclear translocation of p-STAT3 (red) in LOVO cells. Nuclei were counterstained with DAPI (blue). Scale bar: 30 μm. (B) Western blot analysis of phosphorylated STAT3 (p-STAT3, Tyr705) in nuclear fractions of RKO and LOVO cells following PRNP overexpression, with Lamin B1 and β-tubulin serving as nuclear and cytoplasmic loading controls, respectively. (C) Scatter plot of regulatory potential scores for MSN-associated transcription factors from the Cistrome database. STAT3 ranked highest. (D) Dual-luciferase reporter assay measuring MSN promoter activity in LOVO cells with or without STAT3 knockdown. Data are normalized to Renilla luciferase activity. (E) RT-qPCR analysis of MSN mRNA expression after STAT3 knockdown in LOVO cells, normalized to GAPDH. (F-G) Transwell migration and invasion assays demonstrating that STAT3 inhibitor NSC74859 reverses the pro-metastatic effects of PRNP overexpression. Representative images (left) and corresponding quantification (right) are shown.





**Figure S9.** The dynamic internalization of PrPC drives intracellular signal activation.

(A) The internalization of PrPC in RKO and LOVO cells was evaluated using a cleavable cell-surface biotinylation assay. After surface labeling with Sulfo-NHS-SS-Biotin at 4°C, cells were incubated at 37°C for the indicated time intervals to induce endocytosis. Subsequently, the remaining surface-bound biotin was stripped using the reducing agent MesNa. Internalized biotinylated proteins were isolated via Streptavidin pull-down, and the protein levels of internalized PrPC (Pull-down fraction) and total PrPC (Input fraction) were analyzed by Western blotting. β-Tubulin served as a loading control for the Input fraction and as a crucial negative control for the Pull-down fraction. NM, time 0 with no MesNa; MT0, time 0 min with MesNa; MT15, time 15 min with MesNa; MT30, time 30 min with MesNa; MT60, time 60 min with MesNa. (B) Relative quantification of internalized PrPC levels at the indicated time points. (C,E) Subcellular fractionation analysis was performed to evaluate the spatial distribution of PrPC in RKO (C) and LOVO (E) cells treated with or without Dynasore. Na^+^-K^+^-ATPase and β-Tubulin were used as specific markers and loading controls for the plasma membrane and cytoplasmic fractions, respectively. (D,F) Relative quantification of PrPC protein levels in the plasma membrane and cytoplasmic fractions. (G) Western blot analysis of PrPC and phosphorylated STAT3 (p-STAT3, Tyr705) in RKO and LOVO cells treated with or without Dynasore. β-Tubulin was used as a loading control. (H) Relative quantification of the PrPC and p-STAT3 protein levels.


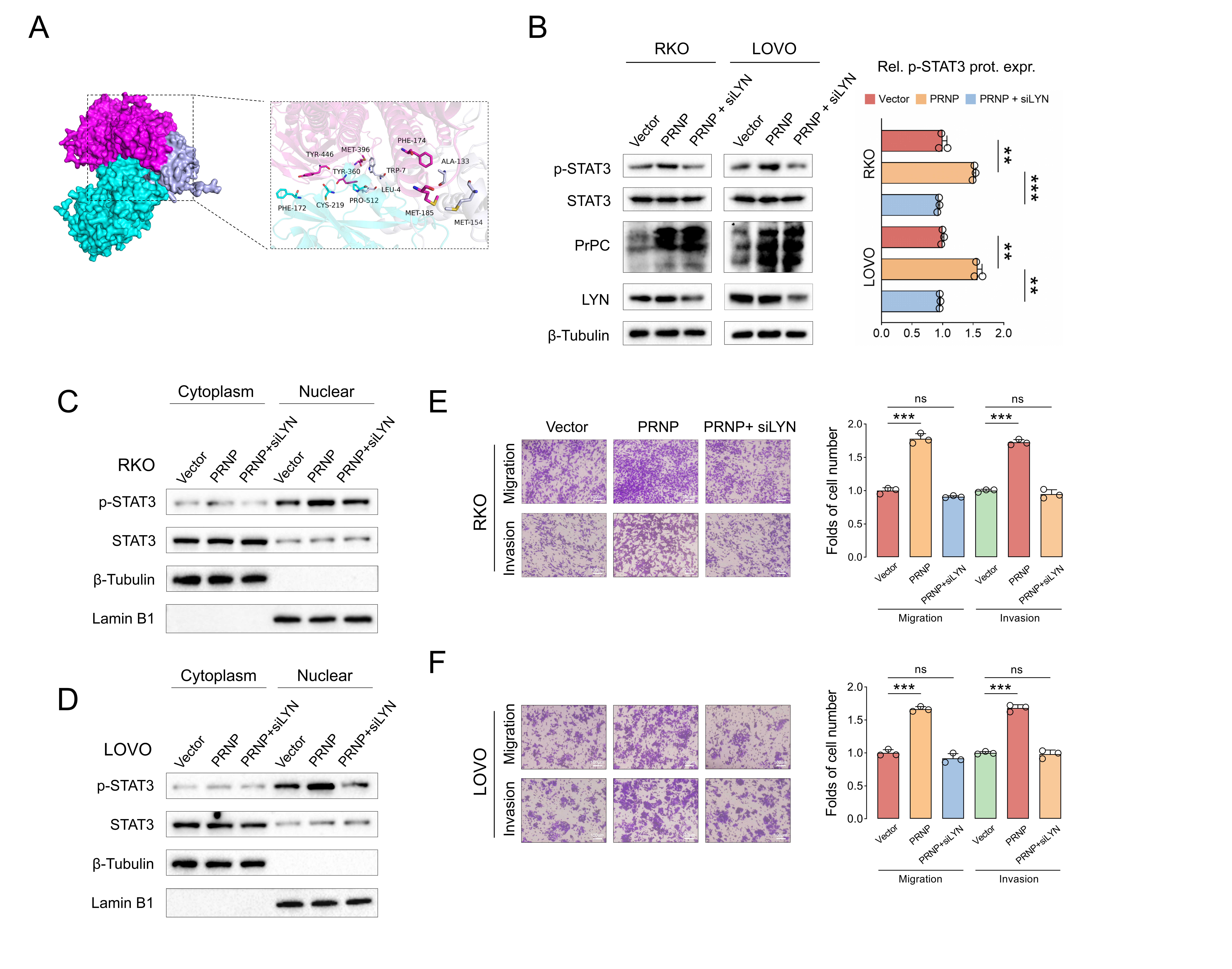


**Figure S10.** PrPC simultaneously interacted with LYN and STAT3 to form a ternary complex.

(A) Computational structural model predicting the ternary complex formation among LYN, STAT3, and PrPC, with hydrophobic interactions identified as a key binding force. (B) Western blot analysis showing that LYN knockdown reverses the PRNP overexpression-induced increase in STAT3 phosphorylation (Tyr705), using β-tubulin as a loading control. (C-D) Subcellular fractionation analysis demonstrates that LYN knockdown attenuates the PrPC-driven nuclear accumulation of p-STAT3 (Tyr705). Lamin B1 and β-tubulin serve as markers for nuclear and cytoplasmic fractions, respectively. (E-F) Transwell migration and invasion assays demonstrating that LYN knockdown rescues the enhanced metastatic phenotype induced by PRNP overexpression.


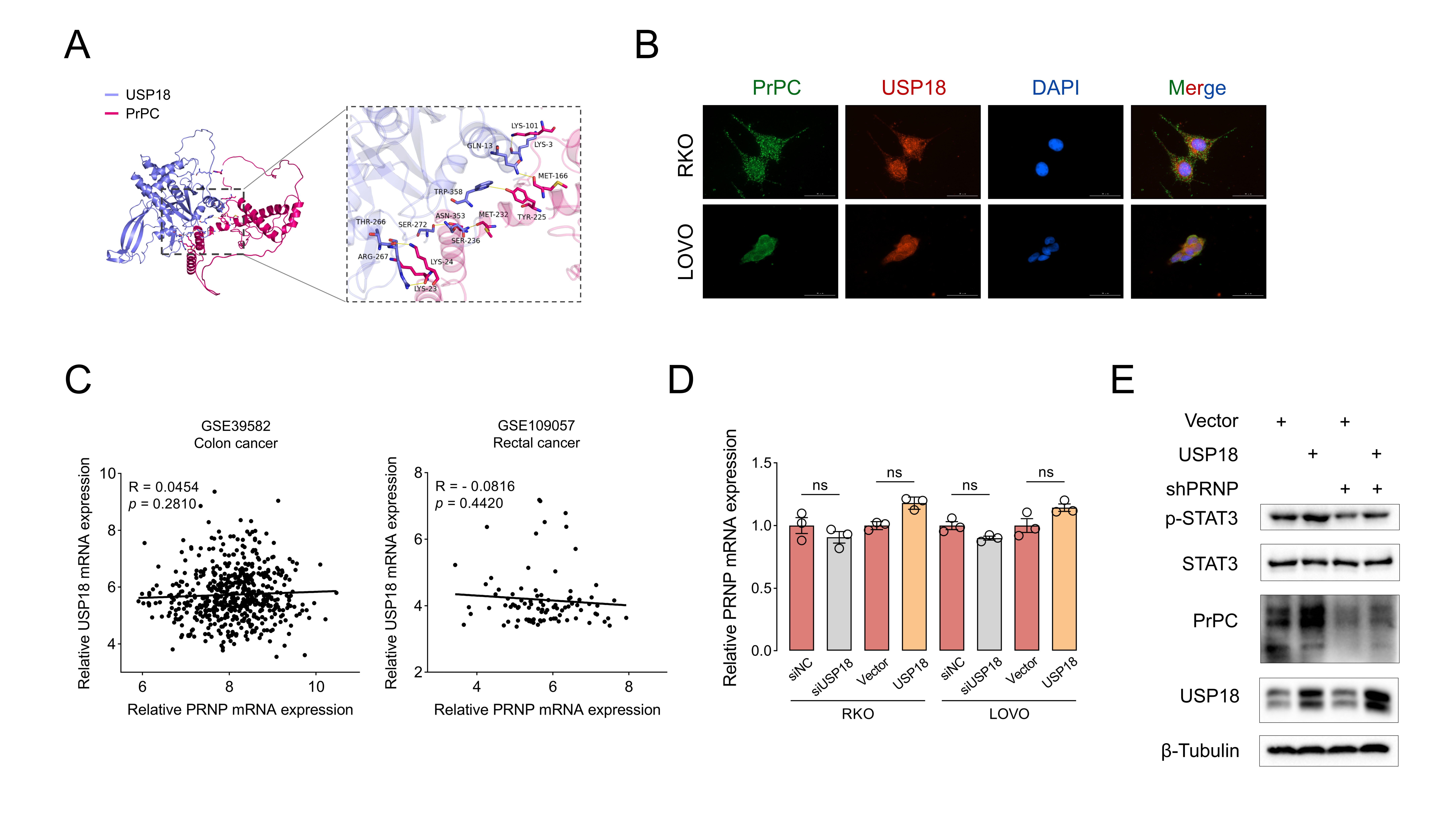


**Figure S11.** USP18 stabilizes PrPC to promote STAT3 phosphorylation in CRC cells.

(A) Predicted structural model of the PrPC-USP18 complex generated by protein-protein docking, suggesting a stable interaction interface. (B) Immunofluorescence staining demonstrates significant co-localization of endogenous PrPC (green) and USP18 (red) in the cytoplasm of RKO and LOVO cells. Nuclei were stained with DAPI (blue). Scale bars: 30 μm. (C) Correlation analysis of PRNP and USP18 mRNA expression in CRC cohorts (GSE39582 and GSE109057). (D) RT-qPCR analysis of PRNP mRNA levels following USP18 knockdown or overexpression, normalized to GAPDH. (E) Western blot analysis of p-STAT3 (Tyr705) levels in RKO cells following genetic perturbations.

**Table S1.** Sequences of shRNA or siRNA

| shRNA | Sequence 5’---3’ |
| --- | --- |
| shPRNP-1 | UCAGUGGAACAAGCCGAGUAA |
| shPRNP-2 | CGACUGCGUCAUAUCACAAU |
| siMSN-1 | AAAUUCGUCAAUGCGCUGC |
| siMSN-2 | UUAGACUGGACAGCAUACG |
| siLYN | GCAUGGAGAAUGGUGGAAA |
| siSTAT3 | GGGACCUGGUGUGAAUUAUTT |
| siUSP18 | CTGCATATCTTCTGGTTTA |

**Table S2.** The primer sequences of qRT-PCR.

| Gene | Forward primer | Reverse primer |
| --- | --- | --- |
| PRNP | AACATGCACCGTTACCCCAA | GACCGTGTGCTGCTTGATTG |
| MSN | CATAGCTCCTGCTCCACAG | GCACAGGCTTCTCTCACCAT |
| USP18 | AAGGTCTTCGGACGGCCTA | GCAGCGAAAACTTGAGGGTAT |

**Table S3.** The information of antibodies.

| Antibodies | SOURCE | IDENTIFIER |
| --- | --- | --- |
| Prion protein | Sigma-aldrich | P0110 |
| STAT3 | CST | 12640 |
| Ecadherin | CST | 3195 |
| Ncadherin | CST | 13116 |
| Vimentin | Proteintech | 10366-1-AP |
| p-STAT3 | CST | 9145 |
| MSN | Abcam | Ab52490 |
| LYN | CST | 2796 |
| USP18 | CST | 4813 |
| β-Tubulin | ZSbio | TA347064 |
| Flag | Abcam | ab205606 |
| His | Proteintech | 66005-1-Ig |
| HA | Proteintech | 51064-2-AP |
| Myc | Proteintech | 60003-2-lg |
| Ubi | Proteintech | 10201-2-AP |

**Table S4. The primer sequences of MSN promoter used in the ChIP assay.**

| Promoter | Forward primer | Reverse primer |
| --- | --- | --- |
| MSN promoter | GAAGAGGTCCTGCTTCAAGG | GGCTGGTGAGGTCTAACTGG |
